# Dual Anti-Inflammatory and Anticancer Activity of Novel 1,5-Diaryl Pyrazole Derivatives: Molecular Modeling, Synthesis, In Vitro Activity, and Dynamics Study

**DOI:** 10.3390/biomedicines12040788

**Published:** 2024-04-03

**Authors:** Priya Deivasigamani, S. M. Esther Rubavathy, Narayanan Jayasankar, Venkatesan Saravanan, Ramasamy Thilagavathi, Muthuramalingam Prakash, Chelliah Selvam, Rajakrishnan Rajagopal, Ahmed Alfarhan, Muthu Kumaradoss Kathiravan, Selvaraj Arokiyaraj, Jesu Arockiaraj

**Affiliations:** 1 Dr APJ Abdul Kalam Research Lab, Department of Pharmaceutical Chemistry, SRM College of Pharmacy, SRM Institute of Science and Technology, Kattankulathur 603203, Chengalpattu District, Tamil Nadu, India; priyad@srmist.edu.in (P.D.); venkatesan_saravanan@srmuniv.edu.in (V.S.); 2 Department of Chemistry, Faculty of Engineering and Technology, SRM Institute of Science and Technology, Kattankulathur 603203, Chengalpattu District, Tamil Nadu, India; es3122@srmist.edu.in (S.M.E.R.);; 3 Department of Pharmacology, SRM College of Pharmacy, SRM Institute of Science and Technology, Kattankulathur 603203, Chengalpattu District, Tamil Nadu, India; narayanj@srmist.edu.in; 4 Department of Biotechnology, Faculty of Engineering, Karpagam Academy of Higher Education, Coimbatore 641021, Tamil Nadu, India; thilagavathir@yahoo.com; 5 Ennam College of Pharmacy, Coimbatore 641032, Tamil Nadu, India; 6 Department of Pharmaceutical Sciences, Joan M. Lafleur College of Pharmacy and Health Sciences, Texas Southern University, Houston, TX 77004, USA; 7 Department of Botany and Microbiology, College of Science, King Saud University, P.O. Box 2455, Riyadh 11451, Saudi Arabia; rrajagopal@ksu.edu.sa (R.R.);; 8 Department of Food Science & Biotechnology, Sejong University, Seoul 05006, Republic of Korea; 9 Department of Biotechnology, Faculty of Science and Humanities, SRM Institute of Science and Technology, Kattankulathur 603203, Chengalpattu District, Tamil Nadu, India

**Keywords:** anti-inflammatory, anticancer, 1,5-diaryl pyrazole, COX enzyme

## Abstract

A series of novel 1,5-diaryl pyrazole derivatives targeting the COX enzyme were designed by combined ligand and structure-based approach. The designed molecules were then further subjected to ADMET and molecular docking studies. Out of 34 designed compounds, the top-10 molecules from the computation studies were synthesized, characterized, and evaluated for COX-2 inhibition and anti-cancer activity. Initially, the target compounds were screened for the protein denaturation assay. The results of the top-five molecules T2, T3, T5, T6, and T9 were further subjected to in vitro COX-2 enzymatic assay and anti-cancer activity. As far as COX-2 inhibitory activity is considered, two compounds, T3 and T5, exhibited the half maximum inhibitory concentration (IC_50_) at 0.781 µM and 0.781 µM respectively. Further, the two compounds T3 and T5, when evaluated for COX-1 inhibition, exhibited excellent inhibitory activity with T3 IC_50_ of 4.655μM and T5 with IC_50_ of 5.596 μM. The compound T5 showed more significant human COX-2 inhibition, with a selectivity index of 7.16, when compared with T3, which had a selectivity index of 5.96. Further, in vitro anti-cancer activity was screened against two cancer cell lines in which compounds T2 and T3 were active against A549 cell lines and T6 was active against the HepG2 cell line. Stronger binding energy was found by comparing MM-PBSA simulations with molecular docking, which suggests that compounds T3 and T5 have a better possibility of being effective compounds, in which T5 showed higher binding affinity. The results suggest that these compounds have the potential to develop effective COX-2 inhibitors as anti-cancer agents.

## 1. Introduction

Prostaglandins play a vital role in the generation of inflammatory responses and act as salient physiological and pathological moderators involved in pain, pyrexia, inflammation, osteoporosis, cancer, male sexual dysfunction, labor, glaucoma, cardiovascular disease, and asthma [1]. Inflammation is involved in the pathogeneses of stroke, arthritis, malignancy, cardiovascular and neurodegenerative diseases, and acts by eliminating the injuring factors such as damaged cells, pathogens, and toxic compounds, followed by initiation of the recovery process [2,3]. During inflammation, the profile and the level of prostaglandin production vary significantly depending on the activity of both the isoforms of cyclooxygenases [4].

Chronic inflammation affects nearly 350 million people worldwide and non-steroidal anti-inflammatory drugs (NSAIDs) are the drugs of choice for pain management. The use of NSAIDs increases from year to year because of the increasing age of the population. But NSAIDs show upper gastrointestinal complications like ulceration, bleeding, and perforation, and are most likely to develop peptic ulcers. This is because cyclooxygenase-1 (COX-1) is generally expressed in the gastrointestinal tract and is accountable for the biosynthesis of the prostaglandins required for platelet aggregation and cytoprotection. On the other hand, cyclooxygenase-2 (COX-2) produces prostaglandins like Prostaglandin E2 (PGE2) and Prostaglandin I2 (PGI2), which are widely known to express cytoprotective effects on the gastrointestinal mucosa by lowering gastric acid production in the stomach by parietal cells, increasing blood flow in the mucosa, and restoring viscous mucus release. COX-2 inhibitors have a minimal effect on the cytoprotective action of PGE2 and PGI2. Hence, they are the most effective anti-inflammatory agents with less toxicity in the gastrointestinal tract because of their selective inhibition of COX-1 and COX-2 sparing action [5,6,7]. 

Selective COX-2 inhibitors increase thrombogenesis, atherosclerosis, and cardiovascular complications due to the inhibition of prostacyclin Prostaglandin I2 (PGI2) production, which is an arachidonic product that opposes the effect of thromboxane. Despite these complications, COX-2 inhibitors are the drugs of preference in conditions like Alzheimer’s disease, Schizophrenia, chronic obstructive pulmonary disease, decrease in synovial inflammation in the case of osteo- and rheumatoid arthritis, viral infections like Dengue, and in various cancers [8,9]. Proteins that bind specifically to COX-2 play a vital task in the promotion of tumorigenesis, and the activity of COX-2 promotes angiogenesis, tissue invasion of tumors, resistance to apoptosis, and chemotherapy. With an increase in immunotherapy, several studies have proven that COX-2 mediates immunosuppression through multiple pathways and COX inhibition might improve the response of immunotherapy [10]. 

Recent evidence suggests the higher expression of COX-2 is involved in the etiology of numerous diseases, like Ankylosing spondylitis, juvenile rheumatoid arthritis, epilepsy, diabetes, Alzheimer’s disease, Parkinson’s disease, schizophrenia, and various types of cancers [8,11]. In solid malignancies, including lung, colon, endometrium, bladder, prostate, pancreas, breast, skin basal, and squamous cell wall, the expression of COX-1 is limited, but COX-2 expression is found to be high, which is associated with neurotoxicity, tumor progression, etc. Inflammatory mediators and reactive nitrogen oxygen species cause the “Nuclear factor kappa B” pathway and “COX-2” to rise, resulting in cancer. COX-2 is responsible for prostanoid production and is triggered by a range of inflammatory stimuli seen in the tumor microenvironment. GPCRs (G-protein-coupled receptors) physiologically mediate the effects of prostanoids that are responsible for cancer progression. COX-2 overexpression is up-regulated in a variety of human malignancies, implying that it plays a significant part in cancer pathogenesis [12,13,14], such as in lung, breast, colorectal, prostate, stomach, and cervical cancers. The angiogenic effect of COX-2 can be blocked by selective COX-2 inhibitors, and this may contribute to a decrease in tumor formation. Hence, targeting the COX-2 enzyme is considered a promising approach for cancer therapy. Also, COX-2 selective inhibitors provide synergistic activity with other antitumor drugs to combat cancer. Studies have also proven that regular use of inhibitors of COX-2 may reduce the threat of cancer by improving the efficacy of chemotherapy and also by preventing multidrug resistance [15,16]. 

Diarylpyrazole substituted with sulphonamide [8,17] is one of the most widely explored pharmacophores for selective COX-2 inhibitors. Celecoxib, Ramifenazone, Rimonabant, and Lonazolac are some of the commercially available pyrazole moieties that are potent COX-2 inhibitors as anticancer agents. We have reported the significance of diversified pyrazoles as COX inhibitors and anti-inflammatories, and against related disorders like cancer in our recent review [18]. Further, we have also reported a thorough quantitative structure–activity relationship (QSAR) study on a series of benzene sulphonamide-substituted 1,5-diaryl pyrazole derivatives, resulting in the identification of the 3D-MoRSEC-6 (atomic charges) and GATSe3 (Sanderson electronegativities) descriptor necessary for selective COX-2 inhibitory activity [19]. Based on the generated QSAR model, new lead molecules were designed and screened virtually, including molecular docking, and dynamics studies, for their COX-2 inhibitory activity. The current work is the continuation to our previous work, which reports a series of novel benzene sulphonamide-substituted 1,5-diaryl pyrazole derivatives as anti-inflammatory and anticancer agents.

## 2. Materials and Methods

### 2.1. Molecular Docking

**Figure 1:**
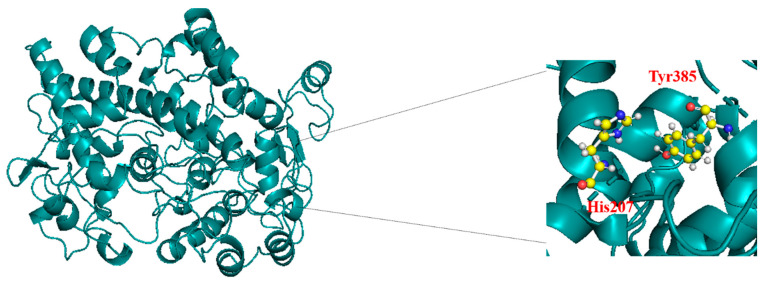
Crystal structure of the protein (PDB ID: 6COX) with its active site of interaction.

The X-ray crystal structure of the protein is retrieved from the Protein Data Bank (PDB). The PDB ID of the protein is 6C0X. The resolution of the protein is 2.80 Å. Figure 1 shows the crystal structure and active sites of the protein. The key residues and active sites of the protein are His207 and Tyr385. The structures of the ligands were optimized by using density functional theory (DFT) based on M06-2X/6-311++G** electronic level of theory. The potential energy surface minima were further verified using frequency calculations (PES). Before docking, the atomic partial charges were computed using the restrained electrostatic potential (RESP) method using an antechamber tool from the Amber package 22. After including the charges and atom types, the protein and the ligand were prepared using AutoDock Tools v4.2.6. The energy-grid boxes and XYZ coordinates were set to the following values: 27.778, 29.389, 40.143 Å, and 126, 86, and 118 Å, respectively. We performed molecular docking using the AutodockVina 1.2.0 [20]. Furthermore, the selected compounds were subjected to molecular dynamics (MD) simulations.

### 2.2. ADMET and Drug-Likeness Filtration

The ADMETs are important characteristics to consider while developing new drugs. In silico toxicology seeks to augment current toxicity assessments by forecasting toxicity, prioritizing compounds, directing toxicity research, and reducing later-stage failures in drug development [21]. There are currently many online and offline resources available to research the potential drug-like properties of synthetic compounds. In the current study, the Swiss ADME online tool is utilized to forecast the drug-likeness feature of the compounds [22]. The Pro Toxicology tool can be used to assess the toxicity parameters [23]. It comprises molecular weight, hydrogen bond (H-bond) donors, acceptors, topological polar surface area, Lipinski rule (drug likeness), hepatotoxicity, and the prediction of the octanol–water partition coefficient (LogP_o/w_). The mentioned criteria predict ADMET characteristics.

### 2.3. MD Simulations

MD simulations were performed for the protein–ligand complexes using Gromacs 2016.3 and Amber [24,25]. The complexes were contained in a 12.0 Å cubic box. Three Na^+^ ions were introduced to the solution to keep it neutral after it was solvated using the TIP3P water model. The calculation is performed using the force-field characteristics of Amber ff14SB for the protein–ligand complex [26]. Amber python was used to convert the amber files into gromacs files. Periodic boundary conditions (PBC) were applied for all three dimensions. The complex file is retrieved from the amber python to perform further MD simulations. To minimize energy, the steepest descent algorithm was employed. After minimization, the system was equilibrated with isothermal–isobaric (NPT) and isothermal–isochoric (NVT) ensembles at 300K and 1 bar of pressure [24]. The Particle Mesh Ewald approach has been applied, with a cutoff distance of 1.2 nm for long-range electrostatic interactions. All bonds containing hydrogen and heavy atoms were limited using the LINCS algorithm integration time steps of 2 fs [27]. The mm–pbsa method was used to calculate the binding free energy of the molecular mechanics/Poisson–Boltzmann surface area (MM–PBSA). Additionally, we conducted a statistical analysis of the MD simulation, looking at factors such as protein–ligand binding parameters, root mean square deviation (RMSD), and root mean square fluctuation (RMSF). Discovery Studio 2021 client [28] and pymol [29] programs were used to view and analyze the trajectory data.

### 2.4. Binding Free-Energy Calculation

It is possible to predict binding free energy using MM–PBSA, even if it produces predictions that are more accurate than typical molecular docking scores and computes more rapidly than other conventional alchemical free-energy calculations. Following MD simulations, this technique was used to ascertain the binding free energy for each of the two protein–ligand complexes. The MM–PBSA approach is used by the tool g_mmpbsa to determine the binding free energy of protein–ligand complexes. The energy components were computed for each system using 100 frames taken from the MD trajectory files. The reciprocal of the grid spacing was set to 1 Å, the external dielectric constant was set to 80, and the internal dielectric constant was set to 2. Other parameters were left at their default values.

### 2.5. Chemical Synthesis 

#### 2.5.1. Chemicals and Solvents

All the essential solvents and chemicals available commercially are procured from Merck, SISCO laboratories, and Sigma-Aldrich and are used without additional purification. For recrystallization, the solvents used are of analytical grade. For performing, TLC (thin-layer chromatography) precoated aluminum sheets (silica gel 60 matrix) are used, and UV light is used as a detecting agent to visualize the spots. The purification of the synthesized compounds was done by column chromatography. A digital melting point apparatus is used for measuring the melting points of the synthesized compounds, which were in powder form. FT-IR spectrophotometer was used for the determination of the IR spectra for the compounds. ^1^H and ^13^C NMR spectra are recorded on an ‘NMR Bruker 400 MHz & 500 MHz’, and (δ) the chemical shifts are expressed in parts per million (ppm) relative to TMS at 0 ppm. Mass spectral analysis was done on an LC–MS spectrometer.

#### 2.5.2. General Procedure for the Synthesis of Compound a to d

Sodium methoxide (40 mmol) was added to 20 mL of methanol and stirred by maintaining the temperature at 0 °C. To this, diethyl oxalate (20 mmol) was added while the solution was still at 0 °C. Acetophenone derivatives (10 mmol) in 10mL of methanol were taken in a beaker and added dropwise to the stirred solution for over an hour and the stirring continued in ice. After complete addition, the solution was allowed to remain at room temperature for a while, and then, it was refluxed for 6 h in an oil bath at 60 to 65 °C. TLC was conducted to monitor the completion of the reaction which is given in Figure S1A. The solution was then left to stand at room temperature overnight and then distilled to remove the solvent. Cool the solution in an ice bath and ice-cold solution of con. HCl was added to adjust the pH to 3. The precipitate obtained was washed 3 times using cold ethanol, and a filter. Recrystallization of the product was done by using 1:1:0.5 of methanol, ethyl acetate, and petroleum ether and later washed with ice water to obtain the intermediate. The purity of the compound was checked using TLC, which was found to be a single spot. 

#### 2.5.3. General Procedure for the Synthesis of Compound e to h

In the first step, compound a to d (15 mmol) was added to 4-Hydrazinyl benzene sulphonamide (15 mmol) in 40 mL of methanol and was refluxed at 60 °C to 80 °C for 6 to 7 h. On checking the completion of the reaction by TLC, the solution was cooled to room temperature. The precipitates are filtered, washed with ethanol, and recrystallized using ethyl acetate, then filtered and dried.

#### 2.5.4. General Procedure for the Synthesis of Compound i to l

Dissolve (6 mmol) of the compounds e to h in ethanol, and after successive addition of hydrazine hydrate (60 mmol), the mixture was stirred for several hours at 80 to 85 °C. TLC was used to monitor the completion of the reaction. On completion, the solution was cooled to room temperature, and a brine solution was added successively to obtain the product. The crude product was filtered and washed with water and with cold ethanol. 

#### 2.5.5. General Procedure for the Synthesis of Compound T1 to T10

Compounds i to l (1 mmol) were dissolved in methanol and various benzaldehydes (1.5 mmol) and 0.5 mL of acetic acid were added and stirred at room temperature for 12 to 14 h. After the reaction was completed, the precipitate was filtered and washed using cold ethanol and water. The target compounds were purified using column chromatogram ethyl acetate and *n* hexane.

### 2.6. In Vitro Anti-Inflammatory Studies

#### 2.6.1. Protein Denaturation Assay

Mizushima and Kobayashi’s technique [21] was employed for the determination of in vitro anti-inflammatory activity by the protein denaturation method for the synthesized compounds. One mL of DMF (DiMethylFormamide) was used to dissolve compounds T1 to T10. Then, a mixture of 500µL of 1% aqueous solution of BSA (bovine albumin serum) and 1.4 mL of phosphate buffer saline (PBS, pH 7.4) was added to 100 µL of varying concentrations of the synthesized compounds 50, 100, and 200 µg/mL. Double-distilled water with a similar volume was taken as the control. The mixtures are then incubated for 15 min at 37 °C and then heated for 10 min at 60 °C. The absorbance was measured at 660nm (SHIMADZU, UV-1800 Spectrophotometer) upon cooling. Diclofenac (100 µg/mL) was used as the reference drug, and the absorbance was measured upon treatment in the same way. The entire experiment was done in triplicate, and the average was calculated.

% inhibition of protein denaturation was calculated by:

$$\%\;inhibition=\frac{\left(Absorbance\;of\;Control-Absorbance\;of\;Sample\right)}{Absorbance\;of\;Control}\times 100$$

#### 2.6.2. In Vitro COX Enzymatic Assay

The ability of the compounds to inhibit COX -2 (IC_50_ values, µM) was determined by using a “COX (human) Inhibitor Screening Assay Kit” procured from Cayman Chemicals (catalog number 701230). With a slight modification in the method as defined in the reference, the COX-2 inhibition assay was performed. Using the percent conversion of arachidonic acid (AA) to PGH_2_, the enzyme activity was calculated. By enzyme immunoassay PGF_2α,_ which is produced from PGH_2_ by reduction with stannous chloride, is calculated. The test compounds’ stock solutions are prepared by dissolving in a minimum amount of DMSO. Human COX-2 (10 µL) in the presence of 10 µL of heme was suspended in 0.1M Tris-HCl buffer (pH 8.0) which contains 2 mM phenol and 5 mM EDTA. This was added to various concentrations of test compounds and, on incubating the solutions at 37 °C for 5 min, 10 µL of AA solution were added. Later, the COX reaction was stopped by adding 1M HCl (50 µL) after 2 min. The intensity of the yellow color which is produced as the product of the enzymatic reaction is determined spectrophotometrically. The percent inhibition was calculated by comparing the compound treated to different control incubations. Celecoxib is utilized as the standard for the assay. The determinations are done in triplicate and the IC_50_ (µM) was determined using the concentration–inhibition response curve.

#### 2.6.3. In Vitro Anticancer Activity

The in vitro anticancer activity is determined by counting the viable cells that are stained with a vital dye. The MTT technique is an accurate measure of the activity of live cells using mitochondrial dehydrogenases. The target tumor cells A549 (human lung adenocarcinoma) and HEPG2 (human liver) cell lines are procured from the “National Centre for Cell Science” (NCCS, Pune). The sources of reagents like Trypsin, DMEM, Pen strip, and FBS are procured from Himedia. Cisplatin is used as the reference. The culture medium was prepared, and the MTT assay was performed as per the general procedure described by Mosmann T. et al., [30] and the % viability of the cells was determined.

## 3. Results and Discussion

### 3.1. QSAR and Docking Studies

Recently we have developed a QSAR model for benzene sulphonamide substituted 1,5-diaryl pyrazole derivatives by multiple linear regression using QSARINS [19]. The 34 novel compounds given in Table S1A were designed based on the key structural requirement for benzene sulphonamide-substituted 1,5-diaryl pyrazole derivatives and were subjected to molecular docking using AutodockVina 4.2.6. inside the COX-2 enzyme active site with Celecoxib as the standard drug followed by ADMET predictions. The structure of the top ten compounds was identified, and the Biovia Discovery Studio Visualizer was used to visualize the binding modes and interactions of the compounds with the amino acid residues in the active site. The top ten compounds exhibited binding free energies ΔGb between −10.80 and −9.69 kcal/mol. Among the ten target compounds, compounds T3 and T5 exhibited the highest affinity with ΔGb of −10.20 and −10.80 kcal/mol, respectively. Almost all the target compounds displayed hydrogen bonding interactions with the sulphonamide group and pyrazole nucleus. The π–π stacking interactions were observed with the phenyl group attached to the pyrazole ring, and the hydrogen bonding interaction with the acylhydrazone showed similar interaction with the standard celecoxib. The interactions exhibited by the ten compounds along with their binding free energies are listed in Table 1.

**Figure 2:**
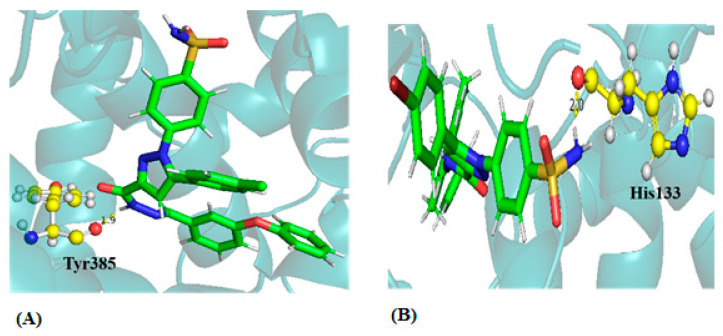
H-bonding interaction of protein–ligand complex after molecular docking (**A**) Compound T3 and (**B**) compound T5.

The docking conformations and interaction sites of the best-docked compounds T3 and T5 are displayed in Figure 2. The compound T3 forms H-bonding interactions with Lys211, Thr212, Gln289, and Tyr385. T3 shows a conventional H-bond (N-H^…^O) with Tyr385 of protein with a distance of 1.9Å, and it also forms π–π stacking interaction with His207, His386, and His388 residues of the protein. Also, compound T5 forms the conventional H-bond (N-H^…^O) with the residue His133 with a distance of ~2.0 Å and a π-–sulfur interaction with the residue Met274. The 3D interactions of the protein–ligand complexes for all the ten compounds are given in Figures S1–S3, providing 2D and 3D interactions of protein–ligand complexes of compounds T3 and T5 after docking.

### 3.2. ADMET and Drug-Likeness Analysis

The ADMET study was performed for the compounds to evaluate how similar these compounds are to the drug molecules. The pharmacokinetic parameters are connected to intestinal permeability and water solubility. It is possible to forecast the drug-like characteristics of particular substances using Lipinski’s rule of five. The molecular weight value indicates the size of the molecule and the drug’s solubility in an aqueous environment is correlated with the molecule’s lipophilicity, which is represented by the logPo/w value. TPSA (topological polar surface area), rotatable bonds, and molar refractivity were employed to research compound drug-like properties in accordance with Veber and Ghose’s guidelines. Further, it is necessary to rule out the compounds with PAINS characteristics in order to prevent false positives. The compounds have been found to have low GI (gastrointestinal tract) adsorption and to be free of hepatotoxicity and carcinogenicity based on the ADMET results identified using Protox II and AdmetSAR. It was clear from the results that the compounds T3 and T5 showed the best binding energy in docking and were found to adhere to drug-like characteristics. The ADMET results for all ten compounds are given in Tables S1–S3.

### 3.3. Synthesis of the Target Compounds

**Figure 3:**
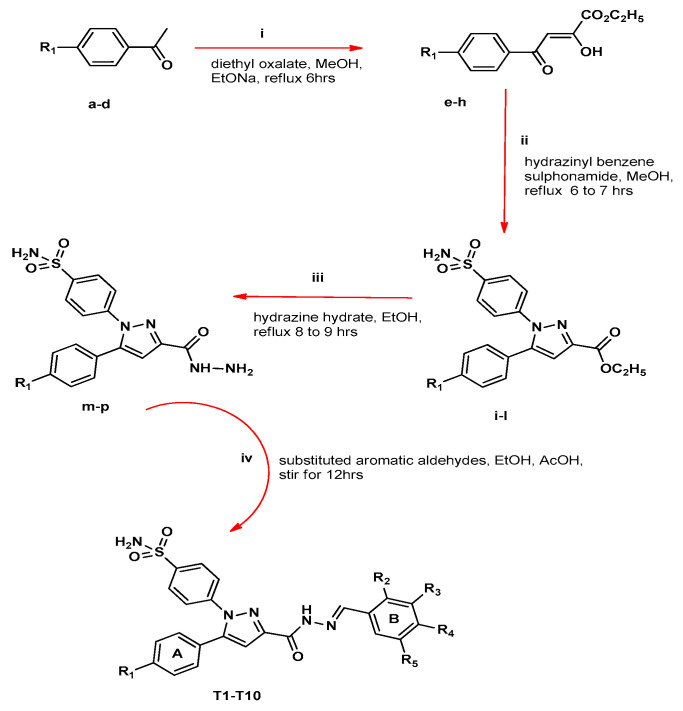
Reagents and conditions: (i) 2.0 equiv diethyl oxalate, MeOH, EtONa, reflux for 6 h, (ii) 1.0 equiv 4-hydrazinyl benzene sulphonamide, MeOH, reflux for 6 h, (iii) 10.0 equiv hydrazine hydrate, EtOH, reflux 8 to 9 h, (iv) 1.5 equiv of substituted aromatic aldehydes, EtOH, AcOH, stir for 12 h.

The synthesis of 1,5-Diarylpyrazoles bearing benzenesulfonamide substituted with phenylacetohydrazide derivatives was carried out as outlined in Figure 3 [31]. Substituted acetophenone derivatives such as chloro and bromomethoxy were reacted with diethyl oxalate in the presence of methanol to yield esters via a claisen condensation reaction. The formed enolate ion undergoes Nu- acyl substitution. The esters were obtained in good yields (85 to 90%), and all were solids with melting points ranging between 385 to 457 °C. IR for the compounds e to h showed stretching for C-O ester at 1138 cm^−1^, confirming the formation of esters, C=C stretching at 1751 cm^−1^ indicated the presence of alkene, C=O stretching at 1680 cm^−1^ confirmed the presence of carbonyl group, and C-OH stretching at 3122 cm^−1^ for the alcoholic group.

The esters were then reacted with 4-hydrazinyl benzene sulphonamide which undergoes cyclization, resulting in the formation of a pyrazole ring. The pyrazole esters were obtained in good yields (80 to 87%), and all were solids with melting points ranging between 195 and 197 °C. The pyrazole esters showed S=O stretching at 1325 cm^−1^, confirming the presence of sulphonamide, C=O stretching for esters at 1645 cm^−1^, C-N stretching at 1149 cm^−1^, and NH stretching in the range of 3097 cm^−1^. The esters were then converted to the corresponding hydrazides upon treatment with hydrazine hydrate, resulting in a 70 to 75% yield. The formation of hydrazide was confirmed by the presence of C=O stretching at 1690 and NH stretching at 3500–3400 cm^−1^. Later, the hydrazides on condensation with substituted aromatic aldehydes gave the corresponding target compounds diaryl pyrazoles with benzenesulphonamide substituted phenylacetohydrazide derivatives. The diaryl pyrazole derivatives were obtained in the range of 55 to 85%, with melting points between 170 and 255 °C. The target compounds were purified by column chromatography using ethyl acetate and *n* hexane, and the purity of the synthesized compounds was found to be in the range of 97 to 99%. The target compounds were soluble in ethanol, DMF, and DMSO, and the compounds were found to be stable. The synthesized compound structures are represented in Table 2. The NMR and MS spectra of the compounds T1–T10 have been included in a supplementary file (Figures S4–S13).

### 3.4. In Vitro Studies

#### 3.4.1. Protein Denaturation Assay

**Figure 4:**
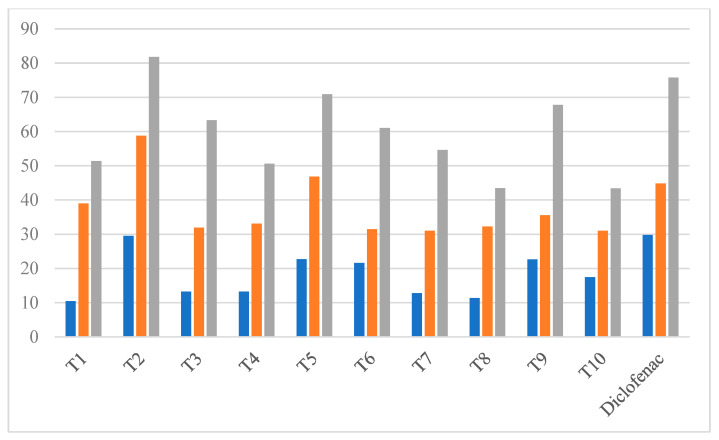
Graphical representation of % inhibition of compounds at concentrations of 50 (blue), 100 (orange), and 200 (grey) µg/mL due to protein denaturation assay.

The anti-inflammatory capacity of the target compound was evaluated using the protein denaturation assay [32]. Compounds T1 to T10 were subjected to a protein albumin denaturation assay at concentrations ranging between 50 and 200 µg/mL using diclofenac as the standard. The graphical representation of % inhibition by protein denaturation assay in Figure 4 clearly shows that, except for the two compounds T8 and T10, all the other compounds were active and demonstrated significant anti-inflammatory activity. Compounds T2, T3, T5, T6, and T9 exhibited the maximum inhibition of albumin denaturation with a % inhibition of 81.77, 63.32, 70.87, 61.05, and 67.77%, respectively, as compared with 75.79% for diclofenac. Compounds T1, T4, T7, T8, and T10 showed less than 50% inhibitory values, showcasing low anti-inflammatory potential when compared with the standard diclofenac. Among the series of ten synthesized compounds, five compounds that were more active toward the protein denaturation assay were subjected to an in vitro COX-2 enzymatic assay. 

#### 3.4.2. In Vitro COX Inhibition Assay

During the initiation of the inflammatory process, the COX enzyme plays a vital role in the progression of inflammatory response. The inflammatory mediators that arise due to the action of COX lead to the chemoattraction of neutrophils, macrophages, eosinophils, etc. Due to the involvement of COX in inflammatory diseases, inhibitors of this enzyme can help in designing effective therapeutic agents [33,34,35]. Hence, the determination of COX inhibition serves as a focal parameter to evaluate the anti-inflammatory potential of the target compounds. This was carried out with the help of a COX (human) inhibitor screening assay kit (Item no. 701230) from Cayman chemicals. On evaluating the in vitro inhibitory action towards both human COX-1 and human COX-2 isoenzymes, the newly synthesized compounds were tested for their ability to specifically inhibit human cyclooxygenase-2 isoenzyme (COX-2). A test substance concentration was considered to be inhibitive when it inhibited human COX-1 or COX-2 by 50%. The compound’s selectivity was measured by its selectivity index values, derived as IC_50_ (COX-1)/IC_50_ (COX-2). 

**Figure 5:**
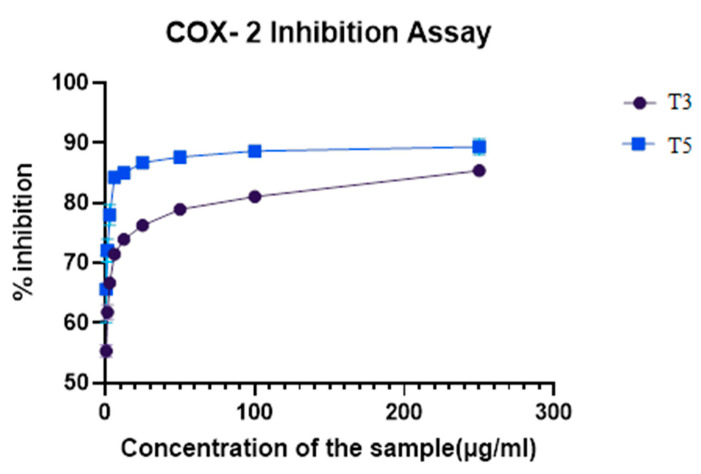
COX-2 inhibition assay of compounds T3 and T5.

**Figure 6:**
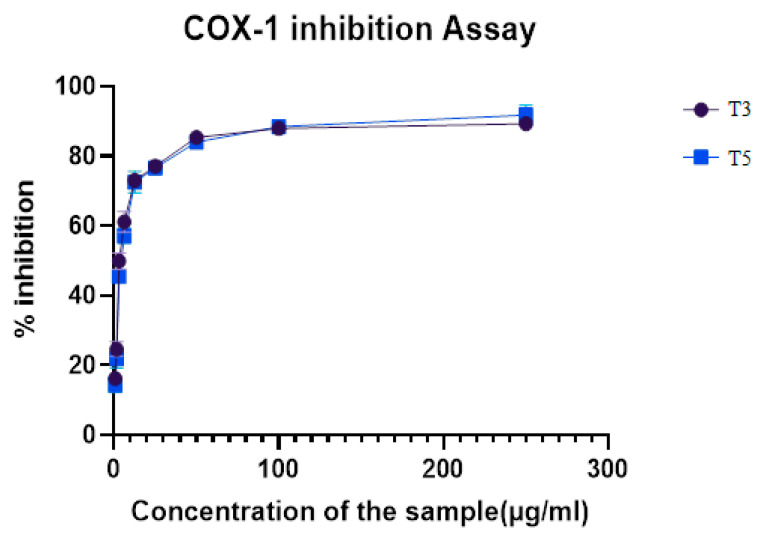
COX-1 inhibition assay of compounds T3 and T5.

COX-2 inhibition for the five target compounds T2, T3, T5, T6, and T9 was performed at various concentrations to determine the percentage of inhibitory activity. The results in Table 3 proved that the compounds exhibited moderate to potent COX-2 inhibition with IC_50_ values between 0.781 and 12 µM. The results of the percentage inhibitory activity of the compounds are depicted in Figure 5. Compounds T3 and T5 having phenoxy and benzyloxy substitution at the acyl hydrazone bridge attached to the pyrazole, with halogens showed a higher % inhibition among the five target compounds. There were no significant differences in the COX-2 inhibitory activity of T3 and T5 since both exhibited half of the maximum inhibitory concentration at 0.781 µM (Tables S4 and S5). The structure–activity relationship suggests that compounds with halogen substitution (Br, Cl) at R^1^ showed better COX-2 inhibition than compounds with methoxy substitution. The bulkier group substitution at the acylhydrazone bridge has better activity. Compound T3 with 4- chloro substitution at R^1^ and –OC_6_H_5_ at R^3^ and compound T5 with 4- bromo substitution at R^1^ and –CH_3_ at R^3^ and R^5^ with –OCH_2_C_6_H_5_ substitution at R^4^ exhibited an IC_50_ of 0. 781 µM. The substitution at the para position of Ring A with electron-withdrawing substituents exhibited excellent COX-2 inhibitory activity compared to the electron-donating substituents. The two compounds T3 and T5 were tested for COX-1 inhibition at various concentrations to determine the percentage of inhibitory activity (Tables S6 and S7). The percentage of inhibitory activity of the compounds is depicted in Figure 6. Thus, among the two compounds, T3 showed more promising COX-1 inhibitory activity than T5.

#### 3.4.3. COX Selectivity Index 

A compound’s selectivity was measured by its selectivity index values, which were derived as IC_50_ (COX-1)/IC_50_ (COX-2). The selective index of compound T3 was 5.96, whereas that for T5 was found to be 7.16. Compound T5 expressed prominent human COX-2 inhibition, with a selectivity index of 7.16 than Compound T3.

#### 3.4.4. In Vitro Anticancer Activity

**Figure 7:**
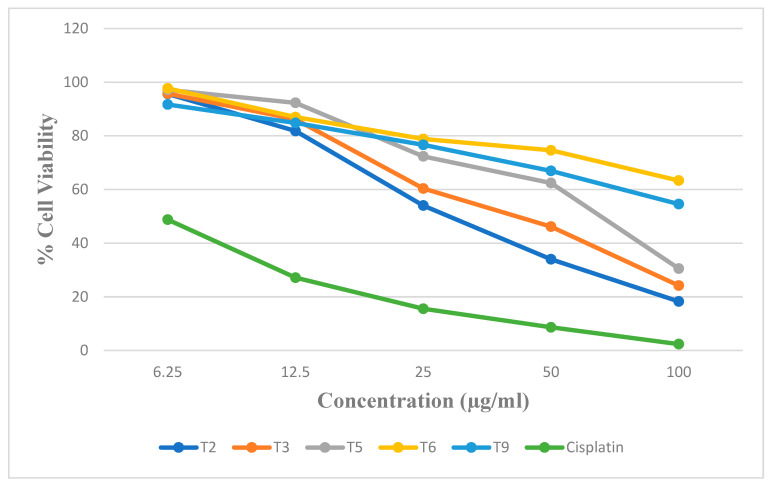
The % cell viability vs. concentration against A549 using MTT assay.

**Figure 8:**
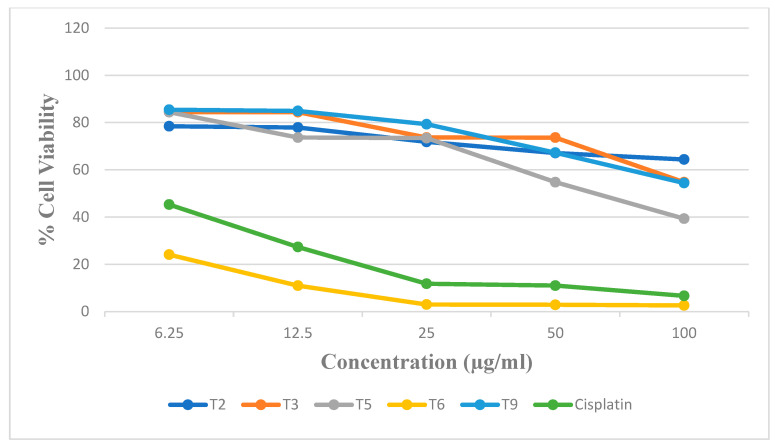
The % of cell viability vs. concentration against HepG2 using MTT assay.

The overexpression of COX-2 in cancer cells has emerged as a potential target for the development of novel anti-cancer drugs [36]. The target compounds tested for in vitro COX-2 were further checked for in vitro anti-cancer activity against A549 (lung cancer) and HepG2 (liver cancer) cell lines using MTT growth inhibition assay. The compounds were tested for cytotoxicity against both the cell lines at concentrations of 6.25, 12.5, 25, 50, and 100 µg/mL. Cisplatin was used as the positive control, and all the tests were done in triplicate. Using the graph where % cell viability is plotted versus concentration, the IC_50_ values are determined with the help of the dose–response regression equation. The % cell viability vs. concentration against A549 and HepG2 cell lines using MTT assay are presented in Table S8, Table S9, Figure 7, and Figure 8, respectively. From the % inhibitory values, it was observed that compound T2 was effective at 25 ug/mL against T3, at 50 µg/mL against A549 cell lines, and Compound T5 was effective at 50 µg/mL against HepG2 cell lines. 

The IC_50_ value for the compounds T2, T3, T5, T6, and T9 and standard (cisplatin) was found to be 31.49, 40.14, 60.95, >100, >100, and 3.97 µM, respectively, against A549. The IC_50_ value for the compounds T2, T3, T5, T6, and T9 and standard (cisplatin) was found to be >100, >100, 66.93, 1.56, >100, and 3.07 µM, respectively, against HepG2. The COX-1 activity was evaluated for only two compounds, T3 and T5, which have shown good COX-2 activity. The compound T6 has a potent COX-2 inhibitory activity at 1.56 µM. The activity of compound T6 may be due to the presence of a high electronegative atom of chlorine at the R^1^ position when compared with T5 having a bromine atom. The activity of compound T6 in the tumor and normal cells will be performed in future studies using in vivo animal models to determine its anti-cancer efficacy. Compound T3 showed potent COX-2 inhibitory activity but weak action on HepG2 viability, and compound T6 has a potent COX-2 inhibitory activity of 1.56 µM, along with potent anti-cancer activity against HepG2 cell lines. Further in vivo correlation between the COX-2 inhibitory potential and anti-cancer activity should be studied in depth to arrive at the conclusion that COX-2 inhibition and anti-cancer activity are related to our compounds. Studies on the immunosuppressive pathway mediated by COX in the case of various cases of cancers, including hepatocellular carcinoma, lung cancer, etc., can be utilized in future studies.

### 3.5. MD Simulations Analysis

**Figure 9:**
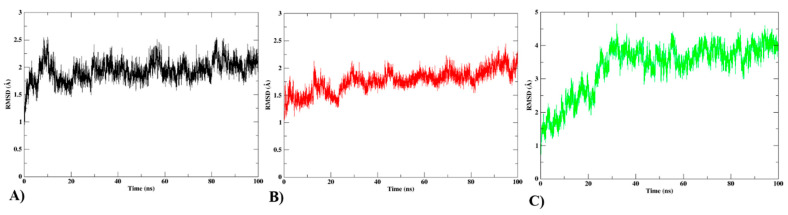
RMSD is calculated for the backbone of (**A**) protein (**B**) protein compound T3, and (**C**) protein Compound T5.

MD simulation was employed to comprehend how protein flexibility and structural alterations impact the interaction profiles of complexes [26,37]. A decreasing RMSD with steady changes throughout the course of the simulation shows that the system has stabilized. Each protein–ligand complex’s RMSD for the backbone was computed. The protein–ligand complexes and protein RMSD plots are shown in Figure 9. The conformational stability of the protein–ligand complex is demonstrated by the average RMSD values of compounds T3 and T5. The average RMSD, RMSF, and Rg of protein–ligand complexes and the average number of H-bonds formed throughout the MD simulation are presented in Tables S10 and S11.

**Figure 10:**
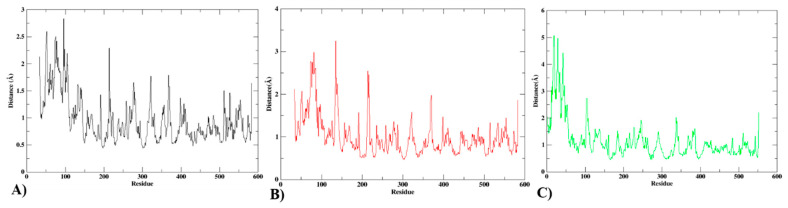
RMSF analysis of Cα atoms. (**A**) Protein, (**B**) protein compound T3, and (**C**) protein compound T5.

Protein Cα atom flexibility is measured using the RMSF. It is a crucial factor in determining a protein–ligand complex’s stability. To achieve a stable conformation that enables a protein–ligand combination to bind strongly, the residues are essential. Each complex’s RMSF was determined and shown on the graph in Figure 10. Overall, the simulation showed that the complex systems had reduced the RMSF profiles, which was consistent with their RMSD profiles.

**Figure 11:**
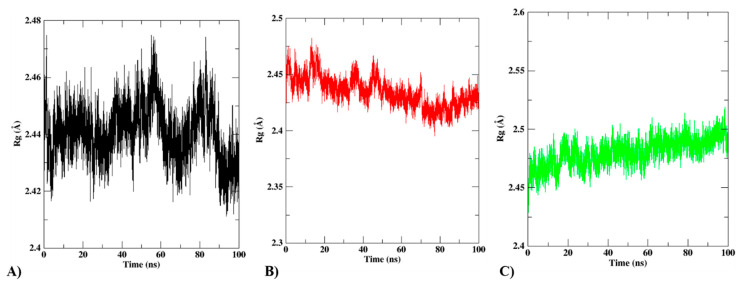
Rg analysis of (**A**) protein, (**B**) protein compound T3, and (**C**) protein compound T5.

The radius of gyration is another tool used to assess the compactness of the protein–ligand complexes (Rg). The Rg can determine how compact the protein structure will be when a protein interacts with a ligand. A high Rg value indicates an unfolded (more compact) protein–ligand interaction. Figure 11 shows that the protein–ligand complexes’ average Rg values vary from 1.8 to 2.0 Å. As a result, each complex shared the same range of Rg values and had an expression of compactness that was substantially equal. According to Figure 11, the free protein and the selected substances preserved the stability and compactness of the complex. 

**Figure 12:**
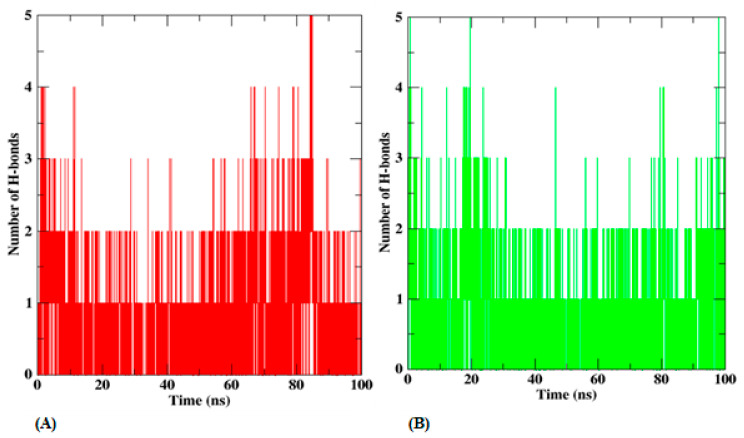
H-bonding interactions of (**A**) protein compound T3 and (**B**) protein compound T5.

The stability of the protein is significantly influenced by the quantity of H-bonded interactions with various substances. The average H-bonding interactions for complexes containing protein and ligand are also shown in Figure 12. Throughout the simulation, T3 interacts with about one H-bond, and other H-bonds that are formed are inconsistent throughout the simulation. Compound T5 forms one H-bond with the protein.

#### Free Energy Calculations (MM-PBSA)

To improve predictions of ligand–protein binding affinity, we used the MM–PBSA approach to predict the free binding energies of the ligands in silico. The MM–PBSA method requires fewer significant in silico calculations than the QM/MM procedures. Van der Waals (vdWs), nonpolar solvation energy, and electrostatic interactions all have negative values in MM–PBSA calculations, indicating their favorable contributions to the binding energy, while polar solvation energy has a positive value, indicating its unfavorable contribution to the binding of the ligand. Using 100 frames of the MD trajectory, the average ΔG (binding free energy) of the two compounds was determined. The average G for compound T3 is calculated by the MM–PBSA to be −20.26 ± 5.01 kcal mol^−1^, while compound T5 has an ΔG of −32.55 ± 3.96 kcal mol^−1^.

Table 4 displays the average estimated binding affinity of natural substances. The top compound T5, showed higher protein-targeted binding affinities. Stronger binding energy was found by comparing MM–PBSA simulations with molecular docking, which suggests that these molecules have a better possibility of being effective. 

From the five synthesized 1,5-diaryl pyrazole derivatives, it is identified that compounds T3 and T5 exhibited better inhibitory activity towards COX-1 isoenzymes. However, T5 showed more significant human COX-2 inhibition. Molecular dynamics studies showed that the compound T5 exhibited higher protein-targeted binding affinities. Hence, further investigation is required to understand the SAR study which could improve the biological activity of the lead compound.

## 4. Conclusions

Based on the binding free energies and ADMET predictions for the compounds designed by QSAR studies, the top-ten compounds were synthesized and confirmed by various spectroscopic studies and had 55 to 85% yield, with melting points ranging between 170 and 261 °C. Compounds T2, T3, T5, T6, and T9 exhibited the maximum inhibition of protein denaturation, among the ten synthesized compounds.

In vitro, COX-2 inhibitory activity revealed that compounds T3 and T5 IC_50_ were at 0.781 µM and 0.781 µM, respectively, as compared to 0.3 µM of standard Celecoxib. The bulkier group substitution at the acylhydrazone bridge was found to improve the activity. Compound T3 with 4- chloro substitution at R^1^ and –OC_6_H_5_ at R^3^ and compound T5 with 4-bromo substitution at R^1^ and –CH_3_ at R^3^ and R^5^ with –OCH_2_C_6_H_5_ substitution at R^4^ exhibited IC_50_ of 0.781 µM. The para-substituted phenyl ring attached to the pyrazole moiety with electron-withdrawing substituents exhibited more potent COX-2 inhibitory activity than those of electron-donating substituents. 

The in vitro inhibitory activity towards COX-1 isoenzymes reported that compound T3 exhibited excellent inhibitory activity, exhibiting IC_50_ of 4.655 μM, and T5 had IC_50_ of 5.596 μM, with a selectivity value of 7.16. The compound T5 showed more significant human COX-2 inhibition than T3, which had a selectivity index of 5.96.

In vitro anti-cancer activity for the target compounds revealed that compounds T2 and T3 were active against A549 cell lines and T6 was active against the HepG2 cell line. Compound T3, which exhibited potent COX-2 inhibitory activity, also showed moderate activity towards the A549 cell line, and compound T6 showed potent anticancer activity against the HepG2 cell line with moderate COX-2 inhibition. Further investigation should be carried out as to whether they have a good relationship between COX-2 inhibition and anti-cancer activity for these compounds. Compound T6, showing significant COX-2 inhibitory potential as well as anti-cancer activity against the HepG2 cell line, can be further investigated using an immunosuppressive pathway mediated through COX. Further modification of compounds T3 and T5 can be done to improve the biological activity. Molecular dynamics studies showed that compound T5 exhibited higher protein-targeted binding affinities. Stronger binding energy was found by comparing MM–PBSA simulations with molecular docking, which suggests that these molecules have a better possibility of being effective. 

### Future Scope

The potential toxicity of the compounds, pharmacokinetic, and pharmacodynamics properties, and in vivo and X-ray diffraction on a single crystal of the synthesized compounds, are to be carried out in the near future.

## Figures and Tables

**Table 1:** Binding energies (in kcal/mol) and H-bonding interactions with amino acid residues in PDB 6COX.

Compound	Binding Energy in kcal/mol	Amino Acid Residues
T1	−9.74	ARG120, CYS36, ASP125
T2	−10.08	LYS333, GLY235, GLY225
T3	−10.20	THR212, TYR385, LYS211, GLN289
T4	−10.18	GLN374
T5	−10.80	ASP325, GLU326, HIS133
T6	−10.12	ASN34, GLY526
T7	−10.02	GLU465
T8	−10.02	SER126, ARG44, ARG120
T9	−10.04	ARG120, TYR130, ASP125
T10	−9.69	ARG120, PRO86, TYR115
Celecoxib	−10.16	ALA527, GLY526, MET522, ARG120

**Table 2:** Structures of the synthesized compounds.

Compound	R^1^	R^2^	R^3^	R^4^	R^5^
T1	OCH_3_	H	OC_6_H_5_	H	H
T2	OCH_3_	H	H	OC_6_H_5_	H
T3	Cl	H	OC_6_H_5_	H	H
T4	Br	H	OC_6_H_5_	H	H
T5	Br	H	CH_3_	OCH_2_C_6_H_5_	CH_3_
T6	Cl	H	CH_3_	OCH_2_C_6_H_5_	CH_3_
T7	Br	H	H	OC_6_H_5_	H
T8	Cl	H	H	OC_6_H_5_	H
T9	Br	OH	H	Cl	H
T10	H	OH	H	Cl	H

**Table 3:** IC_50_ values of selected synthetic compounds by COX-2 and COX-1 enzymatic assay.

Compound	COX-2 IC_50_(µM)	COX-1 IC_50_(µM)	Selectivity Index
T2	12	--	--
T3	0.781	4.655	5.960
T5	0.781	5.596	7.165
T6	1.562	--	--
T9	3.5	--	--
Celecoxib	0.3	--	--

-- Not determined.

**Table 4:** The average binding energy of protein–ligand complexes using MM–PBSA calculation.

Compounds	vdWs	Ele	Pol	SASA	Average Binding Energy in (kcal mol^−1^)
Compound-T3	−56.12	−16.66	58.10	−5.58	−20.26 ± 5.01
Compound-T5	−54.07	−1.58	28.77	−5.67	−32.55 ± 3.96

vdWs—Vander Waals, Ele—ctrostatic solvation, Pol—polar solvation, SASA—solvent-accessible surface area.

## Data Availability

Data will be made available on reasonable request.

## Supplementary Materials

The following supporting information can be downloaded at: https://www.mdpi.com/article/10.3390/biomedicines12040788/s1, Figure S1. Binding interaction of eight compounds from T series with 6COX; Figure S1A. Thin layer chrompatography plate of compounds T3; Figure S2. The 2D interaction of protein-ligand complexes after molecular dockingfor best two compounds (A) T3 and (B) T5; Figure S3. The 3D interaction of protein-ligand complexesafter molecular docking for best two compounds (A) T3 and (B) T5; Figure S4. Compound T1: 4-(5-(4-methoxyphenyl)-3-(2-(3-phenoxybenzylidene) hydrazine-1-carbonyl)-1H-pyrazol-1-yl) benzenesulfonamide. White coloured solid, yield 65%, m.p. 188~190 °C. 3297 (N-H), 3059 (aromatic N-H), 2998 (C–H aromatic), 2840 (aldehyde C–H), 1298 (C-O aromatic ester), 1364 (sulphonamide S=O), 1661 (C=O), 1095 (C-N) and 1439 (alkenes C=C). The 1H NMR (DMSO-d6, 400 MHz) spectra showed δ at 11.61 (s, 1H, CONH), 8.58 (s, 1H, CHN), 7.92 (t, 3H, ArH), 7.70 (d, 2H, ArH), 7.62 (m, 3H, ArH), 7.48 (m, 5H, ArH and SO2NH2), 7.42 (d, 2H, ArH), 7.24 (s, 2H, ArH), 7.11 (s, 1H, CH), 6.98 (d, 2H, ArH), 3.81 (s, 3H, OCH3). 13C NMR δ: 160.60, 159.03, 157.60, 148.56, 146.80, 145.08, 144.06, 142.15, 137.08, 128.68, 127.93, 124.60, 123.79, 123.42, 122.36, 121.82, 121.24, 118.90, 116.54, 114.80, 108.40, 55.74. The mass spectra showed a peak at 568.22 where [M+H]+ is 568.16; Figure S5. Compound T2: 4-(5-(4-methoxyphenyl)-3-(2-(4-phenoxybenzylidene) hydrazine-1-carbonyl)-1H-pyrazol-1-yl) benzenesulfonamide. Pale white coloured solid, yield 68%, m.p. 180~181 °C. 3297 (N-H), 3059 (aromatic N-H), 2998 (C–H aromatic), 2840 (aldehyde C–H), 1278 (C-O aromatic ester), 1365 (sulphonamide S=O), 1661 (C=O),1095(C-N)and 1609 (alkenes C=C). 1H NMR (DMSO-d6, 400 MHz) δ: 11.77 (s, 1H, CONH), 8.53 (s, 1H, CHN), 7.91 (t, 3H, ArH), 7.73 (d, 2H, ArH), 7.57 (m, 3H, CH and SO2NH2), 7.48 (m, 3H, ArH), 7.44 (d, 2H, ArH), 7.14 (m, 3H, ArH), 6.98 (d, 4H, ArH), 3.78 (s, 3H, OCH3) 13C NMR δ:160.22, 159.03, 157.84, 156.26, 147.96, 147.09, 145.08, 144.06, 142.15, 130.70, 129.83, 129.48, 127.17, 126.39, 124.64, 121.53, 119.87, 118.77, 114.79, 108.83, 55.73. The mass spectra showed a peak at 568.20 where [M+H]+ is 568.16; Figure S6. Compound T3: 4-(5-(4-chlorophenyl)-3-(2-(3-phenoxybenzylidene) hydrazine-1-carbonyl)-1H-pyrazol-1-yl) benzenesulfonamide. Light yellow coloured crystals, yield 85%, m.p. 200~202 °C. 3287 (N-H), 3067 (aromatic N-H), 2923 (C–H aromatic), 2851 (aldehyde C–H), 1329 (sulphonamide S=O), 1661 (C=O), 1095 (C-N) and 830 (C-Cl). 1H NMR (DMSO-d6, 400 MHz) δ: 11.90 (s, 1H, CONH), 8.53 (s, 1H, CHN), 7.91 (d, 2H, ArH), 7.59 (t, 3H, ArH), 7.47 (m, 3H, ArH and SO2NH2), 7.35 (m, 3H, ArH), 7.21 (m, 4H, ArH), 7.10 (dd, 4H, ArH), 7.08 (s, 1H, CH). 13C NMR δ: 157.77, 156.77, 148.01, 144.32, 144.01, 141.75, 136.81, 134.46, 131.09, 130.68, 129.39, 128.18, 127.27, 126.49, 124.34, 123.25, 120.82, 119.46, 116.16, 109.70. The mass spectra showed a peak at 572.15 where [M+H]+ is 572.03; Figure S7. Compound T4: 4-(5-(4-bromophenyl)-3-(2-(3-phenoxybenzylidene) hydrazine-1-carbonyl)-1H-pyrazol-1-yl) benzenesulfonamide. Light yellow coloured crystals, yield 84%, m.p. 190~192 °C. 3297 (N-H), 3059 (aromatic N-H), 2838 (C–H aromatic),1364 (sulphonamide S=O), 1660 (C=O),1094(C-N) and 686 (C-Br). 1H NMR (DMSO-d6, 400 MHz) δ: 11.75 (s, 1H, CONH), 8.53 (s, 1H, CHN), 7.91 (d, 2H, ArH), 7.59 (t, 3H, ArH), 7.47 (m, 3H, ArH and SO2NH2), 7.35 (m, 3H, ArH), 7.25 (s, 1H, CH), 7.21 (m, 4H, ArH), 7.10 (d, 2H, ArH), 7.06 (d, 2H, ArH). 13C NMR δ: 157.67, 157.10, 146.81, 143.24, 142.92, 142.01, 141.75, 136.81, 134.46, 131.09, 130.68, 129.39, 128.18, 127.27, 126.49, 124.34, 123.25, 122.34, 121.82, 118.96, 116.56, 108.40. The mass spectra showed a peak at 616.49 where [M+H]+ is 616.06; Figure S8. Compound T5: 4-(3-(2-(4-(benzyloxy)-3,5-dimethylbenzylidene) hydrazine-1-carbonyl)-5-(4-bromophenyl)-1H-pyrazol-1-yl) benzenesulfonamide. Yellow coloured powder, yield 85%, m.p. 225~ 227 °C. 3156 (N-H), 3056 (aromatic N-H), 2938 (C–H aromatic), 1329 (sulphonamide S=O), 1664 (C=O),1014(C-N)and 689 (C-Br). ^1^H NMR (DMSO-d6, 400 MHz) δ: 11.82 (s, 1H, CONH), 8.51 (s, 1H, CHN), 7.90 (d, 2H, ArH), 7.61 (dt, 4H, ArH), 7.51 (d, 2H, SO_2_NH_2_), 7.43 (m, 3H, ArH), 7.35 (m, 3H, ArH), 7.27 (d, 3H, ArH), 4.48 (d, 2H, CH), 2.43 (s, 1H, CH), 2.36 (s, 6H, CH_3_). ^13^C NMR δ: 133.32, 131.83, 131.29, 128.89, 128.54, 128.12, 127.28, 126.52, 122.34, 121.82, 118.96, 116.56, 108.40, 71.4, 16.61. The mass spectra showed a peak at 659.05 where [M^+^H]^+^ is 658.56; Figure S9. Compound T6: 4-(3-(2-(4-(benzyloxy)-3,5-dimethylbenzylidene) hydrazine-1-carbonyl)-5-(4-chlorophenyl)-1H-pyrazol-1-yl)benzenesulfonamide. Light yellow coloured powder, yield 83%, m.p. 235~237 °C. 3156 (N-H), 3064 (aromatic N-H), 2924 (C–H aromatic),1365 (sulphonamide S=O), 1669 (C=O),1024(C-N) and 834 (C-Cl). ^1^H NMR (DMSO-d6, 400 MHz) δ: 11.80 (s, 1H, CONH), 8.45 (s, 1H, CHN), 7.92 (d, 2H, ArH), 7.61 (dt, 4H, ArH), 7.48 (d, 2H, SO_2_NH_2_), 7.41 (m, 3H, ArH), 7.35 (m, 3H, ArH), 7.27 (d, 3H, ArH), 4.48 (d, 2H, CH), 2.43 (s, 1H, CH), 2.30 (s, 6H, CH_3_). ^13^C NMR δ: 133.32, 131.83, 131.29, 128.89, 128.54, 128.12, 127.28, 126.52, 122.34, 121.82, 118.96, 116.56, 108.40, 40.47, 40.34, 39.97, 39.47, 16.61. The mass spectra showed a peak at 614.15 where [M+H]^+^ is 614.12; Figure S10. Compound T7: 4-(5-(4-bromophenyl)-3-(2-(4-phenoxybenzylidene) hydrazine-1-carbonyl)-1H-pyrazol-1-yl) benzenesulfonamide. Yellow coloured solid, yield 80%, m.p. 170~171 °C. 3397 (N-H), 3063 (aromatic N-H), 2998 (C–H aromatic),2840 (aldehyde C–H), 1335 (sulphonamide S=O), 1664 (C=O), 1098 (C-N), 1406 (alkenes C=C) and 689 (C-Br). The ^1^H NMR (DMSO-d6, 400 MHz) spectra showed δ at 11.81 (s, 1H, CONH), 8.55 (s, 1H, CHN), 7.90 (d, 2H, ArH), 7.72 (t, 3H, ArH), 7.60 (m, 3H, ArH), 7.52 (t, 5H, ArH and SO_2_NH_2_), 7.42 (d, 2H, ArH), 7.24 (s, 2H, ArH), 7.11 (s, 1H, CH), 6.98 (d, 2H, ArH). ^13^C NMR δ: 160.24, 157.64, 148.56, 146.74, 145.34, 144.26, 140.15, 135.08, 131.36, 128.08, 127.46, 121.82, 121.24, 118.90, 116.54, 114.80, 108.40. The mass spectra showed a peak at 616.52 where [M+H]^+^ is 616.49; Figure S11. Compound T8: 4-(5-(4-chlorophenyl)-3-(2-(4-phenoxybenzylidene) hydrazine-1-carbonyl)-1H-pyrazol-1-yl) benzenesulfonamide. Light yellow coloured solid, yield 78%, m.p. 190~192 °C. 3397 (N-H), 3065 (aromatic N-H), 2998 (C–H aromatic) 2363 (aldehyde C–H), 1328 (sulphonamide S=O), 1669 (C=O), 1094 (C-N),1409 (alkenes C=C) and 834 (C-Cl). The ^1^H NMR (DMSO-d6, 400 MHz) spectra showed δ at 11.84 (s, 1H, CONH), 8.76 (s, 1H, CHN), 7.88~7.86 (m, 4H, ArH), 7.60 (m, 3H, ArH), 7.48 (s, 2H, SO_2_NH_2_), 7.42 (m, 4H, ArH), 7.24 (t, 4H, ArH), 7.16 (s, 1H, CH), 6.98 (d, 2H, ArH). ^13^C NMR δ: 158.88, 157.60, 148.42, 146.72, 145.28, 144.13, 142.15, 137.08, 129.17, 127.03, 124.60, 123.79, 123.42, 122.36, 116.52, 114.81, 108.87. The mass spectra showed a peak at 572.15 where [M+H]^+^ is 572.03; Figure S12. Compound T9: 4-(5-(4-bromophenyl)-3-(2-(4-chloro-2-hydroxybenzylidene) hydrazine-1-carbonyl)-1H-pyrazol-1-yl) benzenesulfonamide. Yellow coloured crystals, yield 84%, m.p. 250~251 °C. 3282 (O-H), 3436 (N-H), 3186 (aromatic N-H), 3110 (C–H aromatic), 2268 (aldehyde C–H), 1343 (sulphonamide S=O), 1672 (C=O), 1143 (C-N), 1402 (alkenes C=C), 607 (C-Br) and 827 (C-Cl). The ^1^H NMR (DMSO-d6, 400 MHz) spectra showed δ at 12.27 (s, 1H, CONH), 11.21 (s, 1H, OH), 10.23 (s, 1H, CHN), 8.71 (d, 2H, ArH), 7.91~7.89 (m, 2H, ArH), 7.65~7.60 (m, 4H, ArH and SO_2_NH_2_), 7.52 (d, 2H, ArH), 7.33~7.26 (m, 3H, ArH), 6.97 (s, 1H, CH). ^13^C NMR δ: 157.75, 156.50, 146.73, 144.33, 144.09, 141.72, 133.18, 132.31, 131.30, 129.14, 128.48, 127.97, 126.48, 123.46, 123.20, 121.26, 118.96, 118.72, 109.77, 40.47. The mass spectra showed a peak at 573.95 where [M^+^H]^+^ is 574.83; Figure S13. Compound T10: 4-(3-(2-(4-chloro-2-hydroxybenzylidene) hydrazine-1-carbonyl)-5-phenyl-1H pyrazol-1-yl) benzenesulfonamide. Brown coloured solid, yield 55%, m.p. 260~261 °C. 3282 (O-H), 3436 (N-H), 3186 (aromatic N-H), 3110 (C–H aromatic) 2268 (aldehyde C–H), 1343 (sulphonamide S=O), 1672 (C=O), 1143 (C-N), and 1402 (alkenes C=C). The ^1^H NMR (DMSO-d6, 400 MHz) spectra showed δ at 12.24 (s, 1H, CONH), 11.24 (s, 1H, OH), 8.72 (s, 1H, CHN), 7.91 (d, 2H, ArH), 7.89 (m, 3H, ArH), 7.64 (m, 4H, ArH and SO_2_NH_2_), 7.52 (m, 3H, ArH), 7.16 (s, 1H, CH), 6.97 (d, 2H, ArH). ^13^C NMR δ: 157.85, 156.12, 147.74, 145.33, 144.72, 137.18, 132.31, 129.14, 127.04, 126.48, 123.46, 121.26, 118.96, 109.12. The mass spectra showed a peak at 496.05 where [M^+^H]^+^ is 495.95; Table S1. ADME properties calculated using SwissADME; Table S1A. Structure of best designed compounds; Table S2. Toxicological properties calculated using PROTOX-II (IA indicates inactive and A is active); Table S3. Toxicity properties using AdmetSAR; Table S4. % COX-2 Inhibition of compound T3; Table S5. % COX-2 Inhibition of compound T5; Table S6. % COX-1 Inhibition of compound T3; Table S7. % COX-1 Inhibition of compound T5; Table S8. % cell viability of selected synthetic compounds against A549 cell line; Table S9. Cell viability of selected synthetic compounds against HepG2 cell lines; Table S10. The average values of RMSD, RMSF, and Rgof protein-ligand complexes; Table S11. The average number of H-bonds formed throughout the MD simulation.
