# Supplementary material for: Dual Anti-Inflammatory and Anticancer Activity of Novel 1,5-Diaryl Pyrazole Derivatives: Molecular Modeling, Synthesis, In Vitro Activity, and Dynamics Study
Source: Biomedicines. 2024 Apr 3;12(4):788. doi: 10.3390/biomedicines12040788 (PMC11048033; doi:10.3390/biomedicines12040788)
Supplement: Supplementary file 1 [file biomedicines-12-00788-s001.zip › biomedicines-2892895-supplementary.pdf]

E-Supplementary File

Dual Anti-Inflammatory and Anticancer Activity of Novel 1,5-Diaryl Pyrazole Derivatives: Molecular Modeling, Synthesis, In Vitro Activity, and Dynamics Study

Priya Deivasigamani [1], S. M. Esther Rubavathy [2], Narayanan Jayasankar[3], Venkatesan Saravanan[1], Ramasamy Thilagavathi [4], Muthuramalingam Prakash [2], Chelliah Selvam [5], Rajakrishnan Rajagopal [6], Ahmed Alfarhan [6], Muthu Kumaradoss Kathiravan [1,*], Selvaraj Arokiyaraj [7,*] and Jesu Arockiaraj [8]

1 Dr APJ Abdul Kalam Research Lab, Department of Pharmaceutical Chemistry, SRM College of Pharmacy, SRM Institute of Science and Technology, Kattankulathur 603203, Chengalpattu District, Tamil Nadu, India; priyad@srmist.edu.in (D.P.); venkatesan_saravanan@srmuniv.edu.in (S.V.)

2 Department of Chemistry, Faculty of Engineering and Technology, SRM Institute of Science and Technology, Kattankulathur 603203, Chengalpattu District, Tamil Nadu, India; es3122@srmist.edu.in (S.M.E.R.); prakashm4@srmist.edu.in (M.P.)

3 Department of Pharmacology, SRM College of Pharmacy, SRM Institute of Science and Technology, Kattankulathur 603203, Chengalpattu District, Tamil Nadu, India; narayanj@srmist.edu.in

4 Department of Biotechnology, Faculty of Engineering, Karpagam Academy of Higher Education, Coimbatore 641021, Tamil Nadu, India & Ennam College of Pharmacy, Coimbatore 641032; thilagavathir@yahoo.com

5 Department of Pharmaceutical Sciences, Joan M. Lafleur College of Pharmacy and Health Sciences, Texas Southern University, Houston, TX 77004, USA; cselvam@yahoo.com

6 Department of Botany and Microbiology, College of Science, King Saud University, P.O. Box 2455, Riyadh 11451, Saudi Arabia; rrajagopal@ksu.edu.sa (R.R.); alfarhan@ksu.edu.sa (A.A.)

7 Department of Food Science & Biotechnology, Sejong University, Seoul 05006, Republic of Korea

8 Department of Biotechnology, Faculty of Science and Humanities, SRM Institute of Science and Technology, Kattankulathur 603203, Chengalpattu District, Tamil Nadu, India; jesuaroa@srmist.edu.in

* Correspondence: kathirak@srmist.edu.in (M.K.K.); arokiyaraj16@sejong.ac.kr (S.A.)

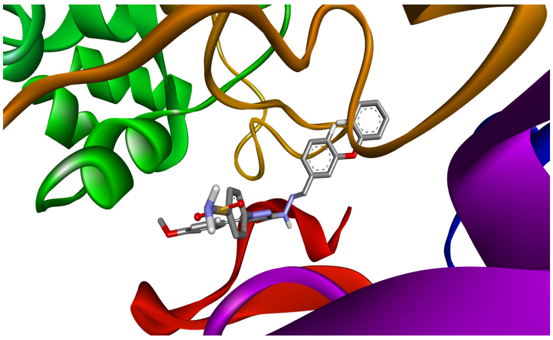

Compound T1

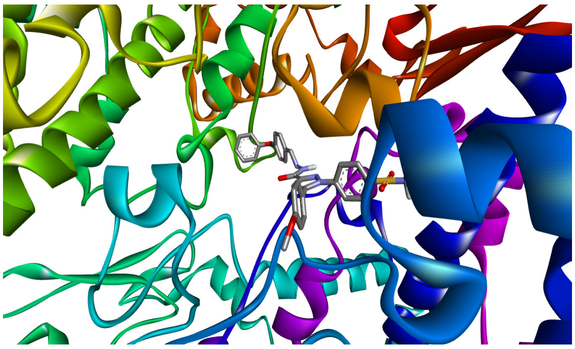

Compound T2

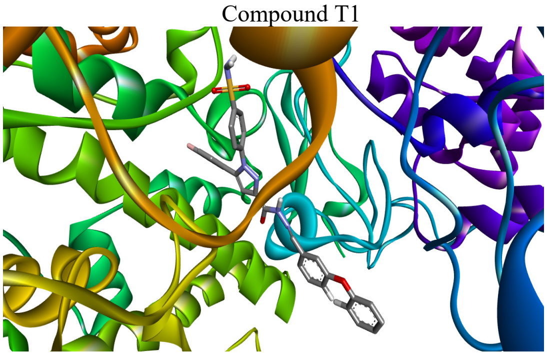

Compound T4

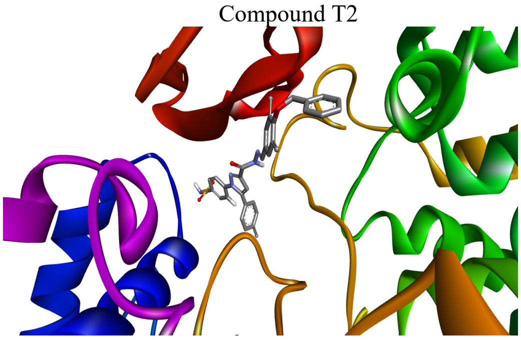

Compound T6

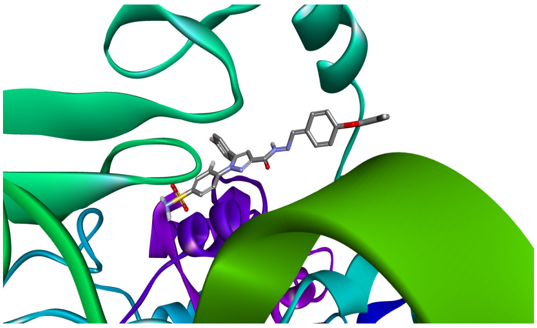

Compound T7

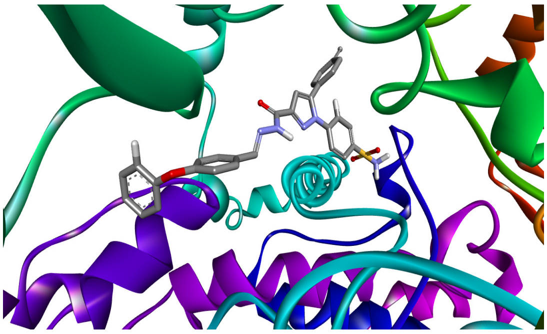

Compound T8

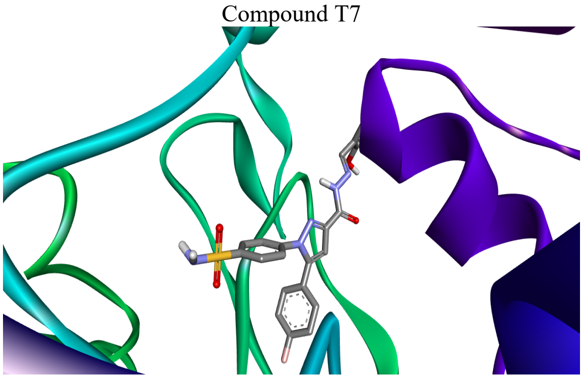

Compound T9

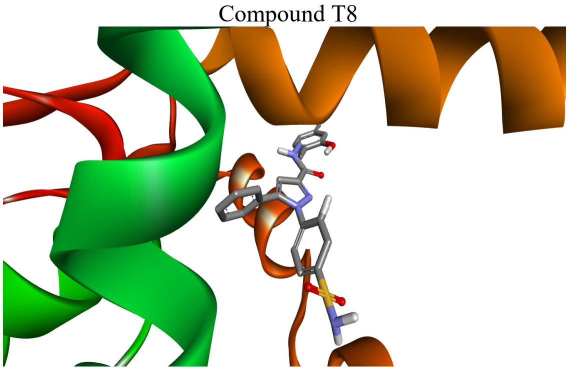

Compound T10

Figure S1. Binding interaction of eight compounds from T series with 6COX

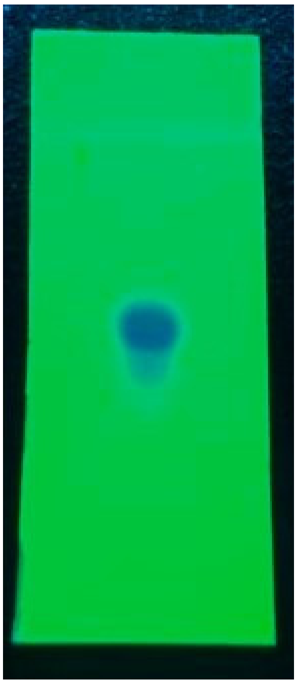

Figure S1A. Thin layer chromatography plate of compounds T3

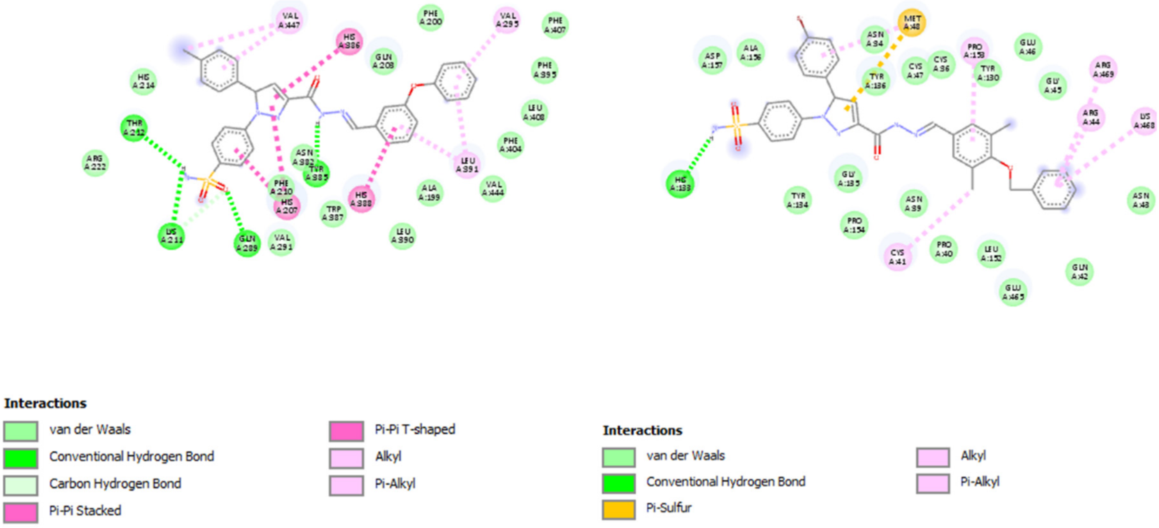

A) **B)**

Figure S2. The 2D interaction of protein-ligand complexes after molecular dockingfor best two compounds **A)** T3 and **B)** T5

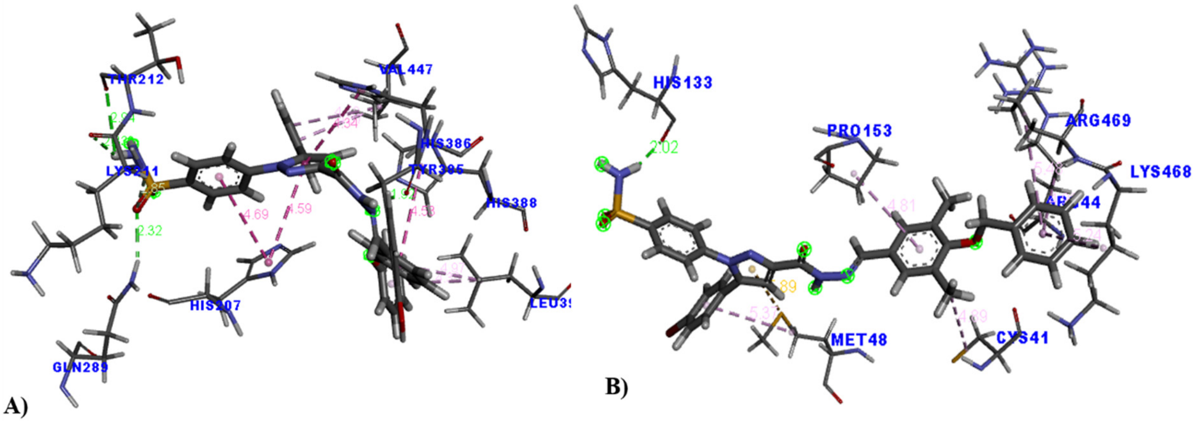

Figure S3. The 3D interaction of protein-ligand complexesafter molecular docking for best two compounds **A)** T3 and **B)** T5

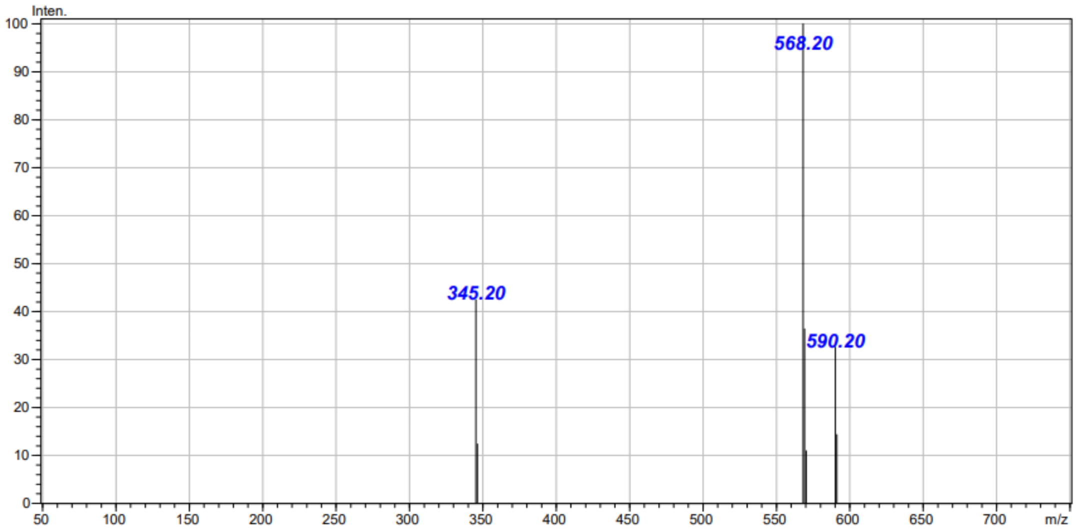

Figure. S4 Compound T1*:* 4-(5-(4-methoxyphenyl)-3-(2-(3-phenoxybenzylidene) hydrazine-1-carbonyl)-1H-pyrazol-1-yl) benzenesulfonamide. White coloured solid, yield 65%, m.p. 188~ 190°C. 3297 (N-H), 3059 (aromatic N-H), 2998 (C–H aromatic), 2840 (aldehyde C – H), 1298 (C-O aromatic ester), 1364 (sulphonamide S=O), 1661 (C=O), 1095 (C-N) and 1439 (alkenes C=C). The ^{1}H NMR (DMSO-d6, 400 MHz) spectra showed δ at 11.61 (s, 1H, CONH), 8.58 (s, 1H, CHN), 7.92 (t, 3H, ArH), 7.70 (d, 2H, ArH), 7.62 (m, 3H, ArH), 7.48 (m, 5H, ArH and SO_2NH_2), 7.42 (d, 2H, ArH), 7.24 (s, 2H, ArH), 7.11 (s, 1H, CH), 6.98 (d, 2H, ArH), 3.81 (s, 3H, OCH3). ^{13}C NMR δ: 160.60, 159.03, 157.60, 148.56, 146.80, 145.08, 144.06, 142.15, 137.08, 128.68, 127.93, 124.60, 123.79, 123.42, 122.36, 121.82, 121.24, 118.90, 116.54, 114.80, 108.40, 55.74. The mass spectra showed a peak at 568.22 where $[M+H]^+$ is 568.16.

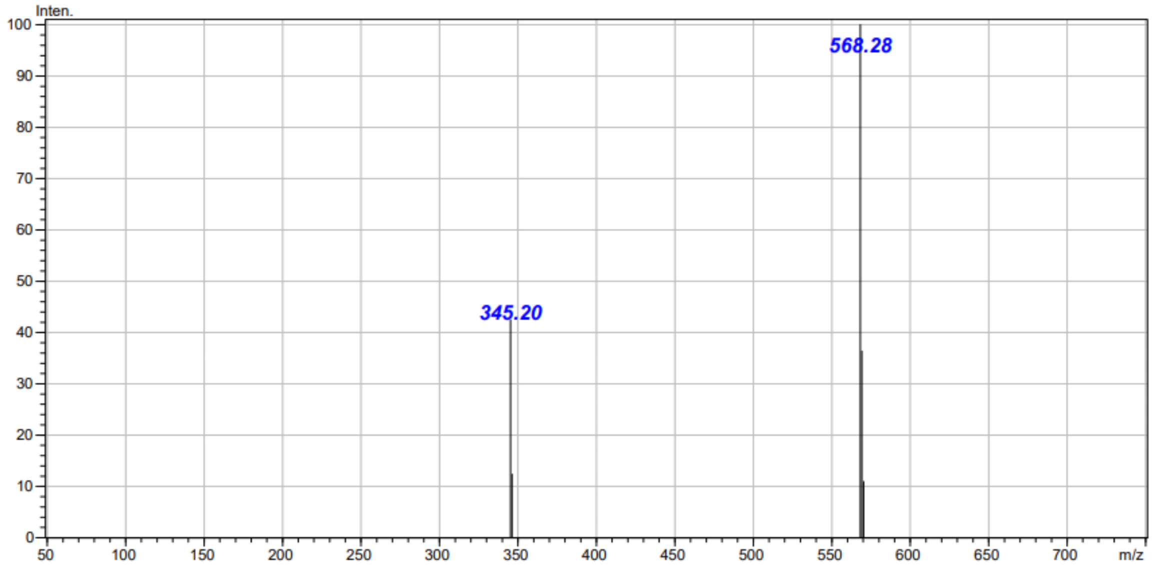

Figure. S5 Compound T2: 4-(5-(4-methoxyphenyl)-3-(2-(4-phenoxybenzylidene) hydrazine-1-carbonyl)-1H-pyrazol-1-yl) benzenesulfonamide. Pale white coloured solid, yield 68%, m.p. 180~ 181°C. 3297 (N-H), 3059 (aromatic N-H), 2998 (C – H aromatic),2840(aldehyde C – H), 1278 (C-O aromatic ester), 1365 (sulphonamide S=O), 1661 (C=O),1095(C-N)and 1609 (alkenes C=C). ^{1}H NMR (DMSO-d6, 400 MHz) δ: 11.77 (s, 1H, CONH), 8.53 (s, 1H, CHN), 7.91 (t, 3H, ArH), 7.73 (d, 2H, ArH), 7.57 (m, 3H, CH and SO_2NH_2), 7.48 (m, 3H, ArH), 7.44 (d, 2H, ArH), 7.14 (m, 3H, ArH), 6.98 (d, 4H, ArH), 3.78 (s, 3H, OCH3) ^{13}C NMR δ:160.22, 159.03, 157.84, 156.26, 147.96, 147.09, 145.08, 144.06, 142.15, 130.70, 129.83, 129.48, 127.17, 126.39, 124.64, 121.53, 119.87, 118.77, 114.79, 108.83, 55.73. The mass spectra showed a peak at 568.20 where $[M+H]^+$ is 568.16.

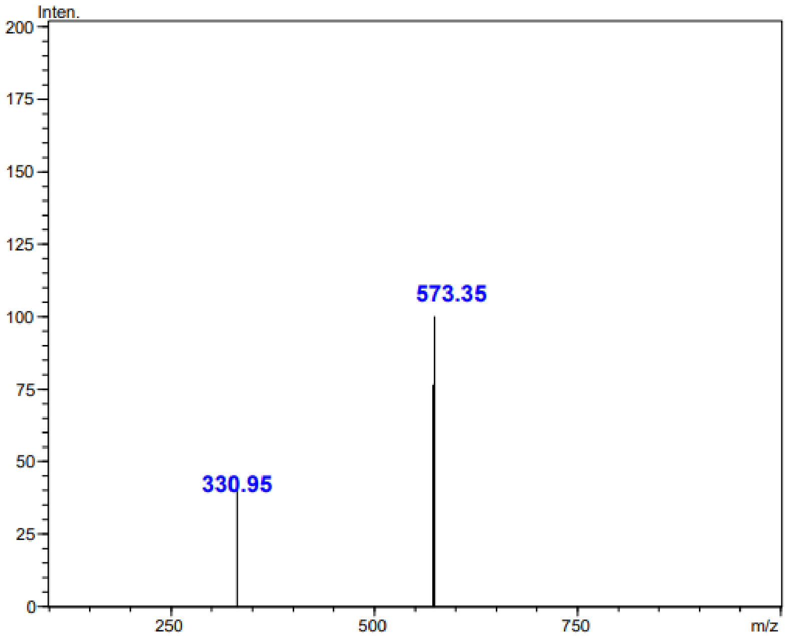

Figure. S6 Compound T3: 4-(5-(4-chlorophenyl)-3-(2-(3-phenoxybenzylidene) hydrazine-1-carbonyl)-1H-pyrazol-1-yl) benzenesulfonamide. Light yellow coloured crystals, yield 85%, m.p. 200~ 202°C. 3287 (N-H), 3067 (aromatic N-H), 2923 (C–H aromatic),2851 (aldehyde C – H), 1329 (sulphonamide S=O), 1661 (C=O),1095 (C-N) and 830 (C-Cl). ^{1}H NMR (DMSO-d6, 400 MHz) δ: 11.90 (s, 1H, CONH), 8.53 (s, 1H, CHN), 7.91 (d, 2H, ArH), 7.59 (t, 3H, ArH), 7.47 (m, 3H, ArH and SO_2NH_2), 7.35 (m, 3H, ArH), 7.21 (m, 4H, ArH), 7.10 (dd, 4H, ArH), 7.08 (s, 1H, CH). ^{13}C NMR δ: 157.77, 156.77, 148.01, 144.32, 144.01, 141.75, 136.81, 134.46, 131.09, 130.68, 129.39, 128.18, 127.27, 126.49, 124.34, 123.25, 120.82, 119.46, 116.16, 109.70. The mass spectra showed a peak at 572.15 where [M+H]+ is 572.03.

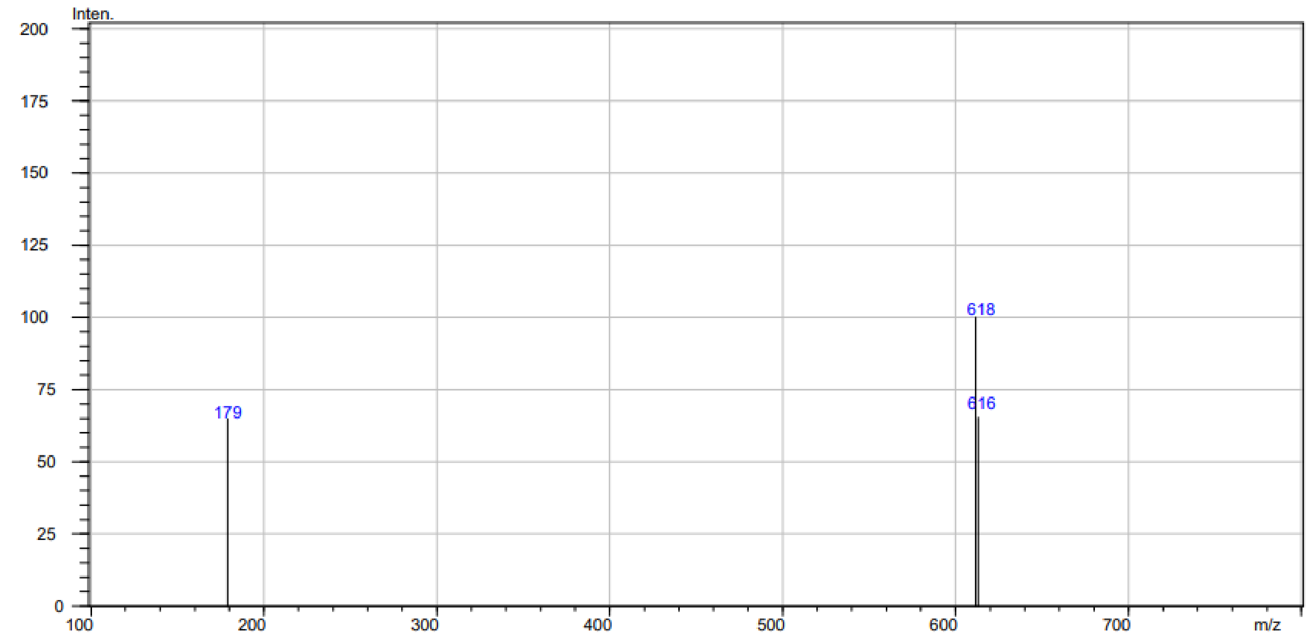

Figure. S7 Compound T4: 4-(5-(4-bromophenyl)-3-(2-(3-phenoxybenzylidene) hydrazine-1-carbonyl)-1H-pyrazol-1-yl) benzenesulfonamide. Light yellow coloured crystals, yield 84%, m.p. 190~ 192°C.3297 (N-H), 3059 (aromatic N-H), 2838 (C – H aromatic),1364 (sulphonamide S=O), 1660 (C=O),1094(C-N) and 686 (C-Br). ^{1}H NMR (DMSO-d6, 400 MHz) δ: 11.75 (s, 1H, CONH), 8.53 (s, 1H, CHN), 7.91 (d, 2H, ArH), 7.59 (t, 3H, ArH), 7.47 (m, 3H, ArH and SO_2NH_2), 7.35 (m, 3H, ArH), 7.25 (s, 1H, CH), 7.21 (m, 4H, ArH), 7.10 (d, 2H, ArH), 7.06 (d, 2H, ArH). ^{13}C NMR δ: 157.67, 157.10, 146.81, 143.24, 142.92, 142.01, 141.75, 136.81, 134.46, 131.09, 130.68, 129.39, 128.18, 127.27, 126.49, 124.34, 123.25, 122.34, 121.82, 118.96, 116.56, 108.40. The mass spectra showed a peak at 616.49 where $[M+H]^+$ is 616.06.

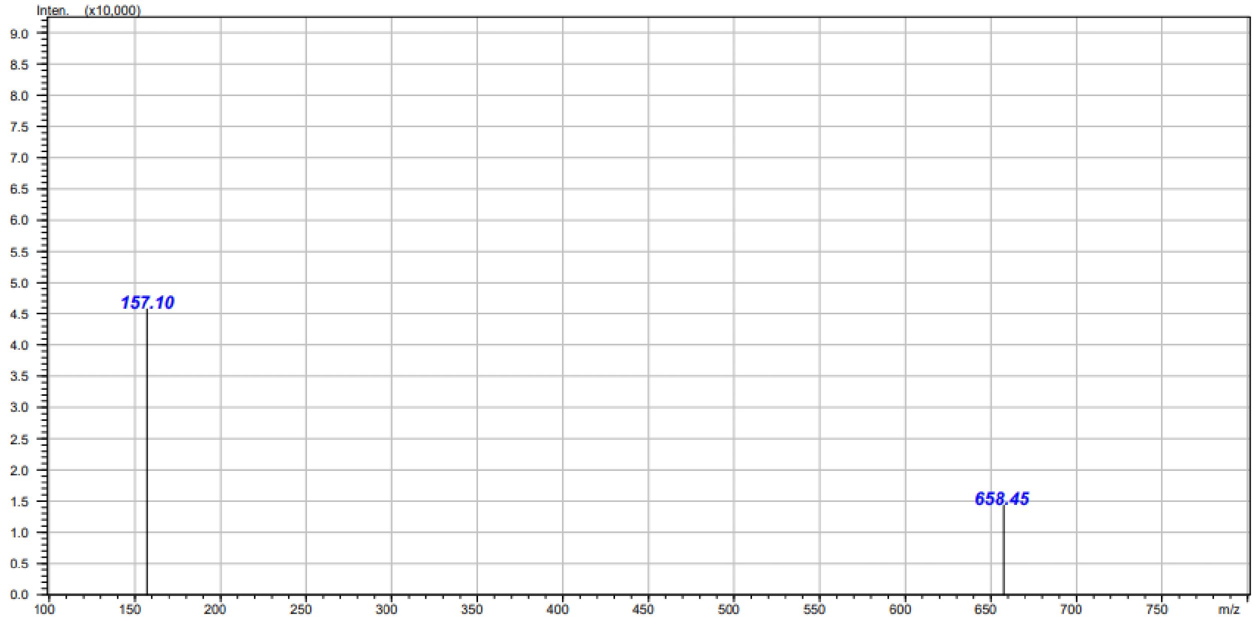

Figure. S8 Compound T5: 4-(3-(2-(4-(benzyloxy)-3,5-dimethylbenzylidene) hydrazine-1-carbonyl)-5-(4-bromophenyl)-1H-pyrazol-1-yl) benzenesulfonamide. Yellow coloured powder, yield 85%, m.p. 225~ 227°C. 3156 (N-H), 3056 (aromatic N-H), 2938 (C–H aromatic),1329 (sulphonamide S=O), 1664 (C=O),1014(C-N)and 689 (C-Br). ^{1}H NMR (DMSO-d6, 400 MHz) δ: 11.82 (s, 1H, CONH), 8.51 (s, 1H, CHN), 7.90 (d, 2H, ArH), 7.61 (dt, 4H, ArH), 7.51 (d, 2H, SO_2NH_2), 7.43 (m, 3H, ArH), 7.35 (m, 3H, ArH), 7.27 (d, 3H, ArH), 4.48 (d, 2H, CH), 2.43 (s, 1H, CH), 2.36 (s, 6H, CH_3). ^{13}C NMR δ: 133.32, 131.83, 131.29, 128.89, 128.54, 128.12, 127.28, 126.52, 122.34, 121.82, 118.96, 116.56, 108.40, 71.4, 16.61. The mass spectra showed a peak at 659.05 where $[M^{+}H]^{+}$ is 658.56

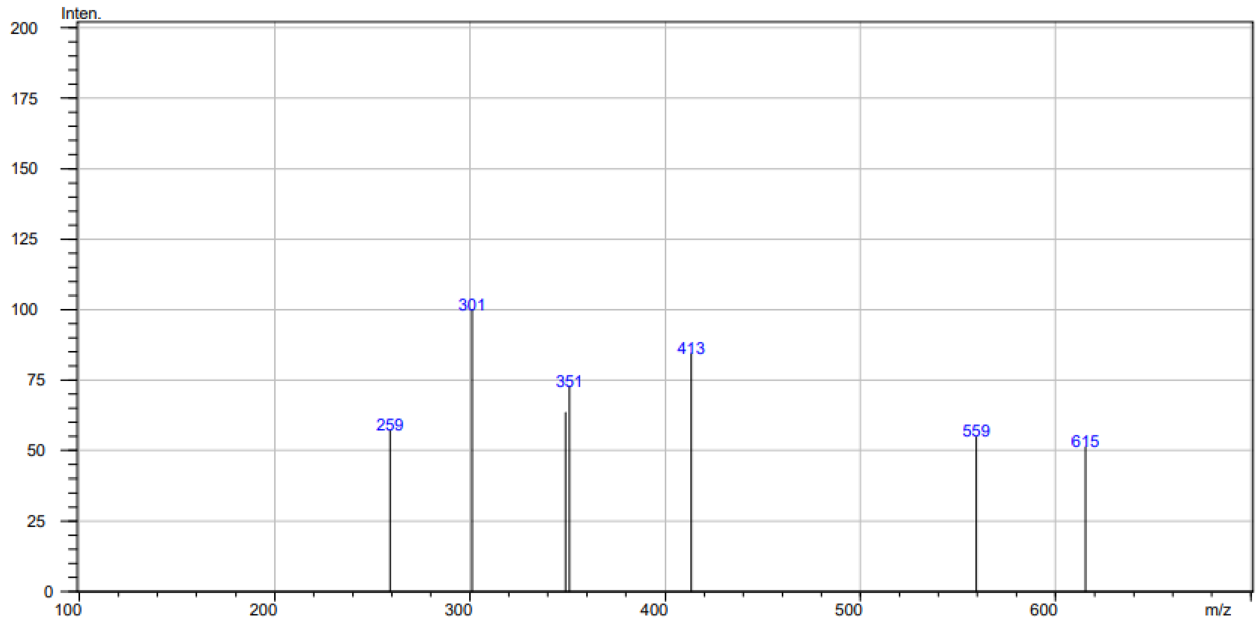

Figure. S9 Compound T6: 4-(3-(2-(4-(benzyloxy)- 3,5-dimethylbenzylidene) hydrazine-1-carbonyl)-5-(4-chlorophenyl)-1H-pyrazol-1-yl)benzenesulfonamide. Light yellow coloured powder, yield 83%, m.p. 235~ 237°C. 3156 (N-H), 3064 (aromatic N-H), 2924 (C–H aromatic),1365 (sulphonamide S=O), 1669 (C=O),1024(C-N)and 834 (C-Cl). ^{1}H NMR (DMSO-d6, 400 MHz) δ: 11.80 (s, 1H, CONH), 8.45 (s, 1H, CHN), 7.92 (d, 2H, ArH), 7.61 (dt, 4H, ArH), 7.48 (d, 2H, SO_2NH_2), 7.41 (m, 3H, ArH), 7.35 (m, 3H, ArH), 7.27 (d, 3H, ArH), 4.48 (d, 2H, CH), 2.43 (s, 1H, CH), 2.30 (s, 6H, CH_3). ^{13}C NMR δ: 133.32, 131.83, 131.29, 128.89, 128.54, 128.12, 127.28, 126.52, 122.34, 121.82, 118.96, 116.56, 108.40, 40.47, 40.34, 39.97, 39.47, 16.61. The mass spectra showed a peak at 614.15 where $[M+H]^+$ is 614.12

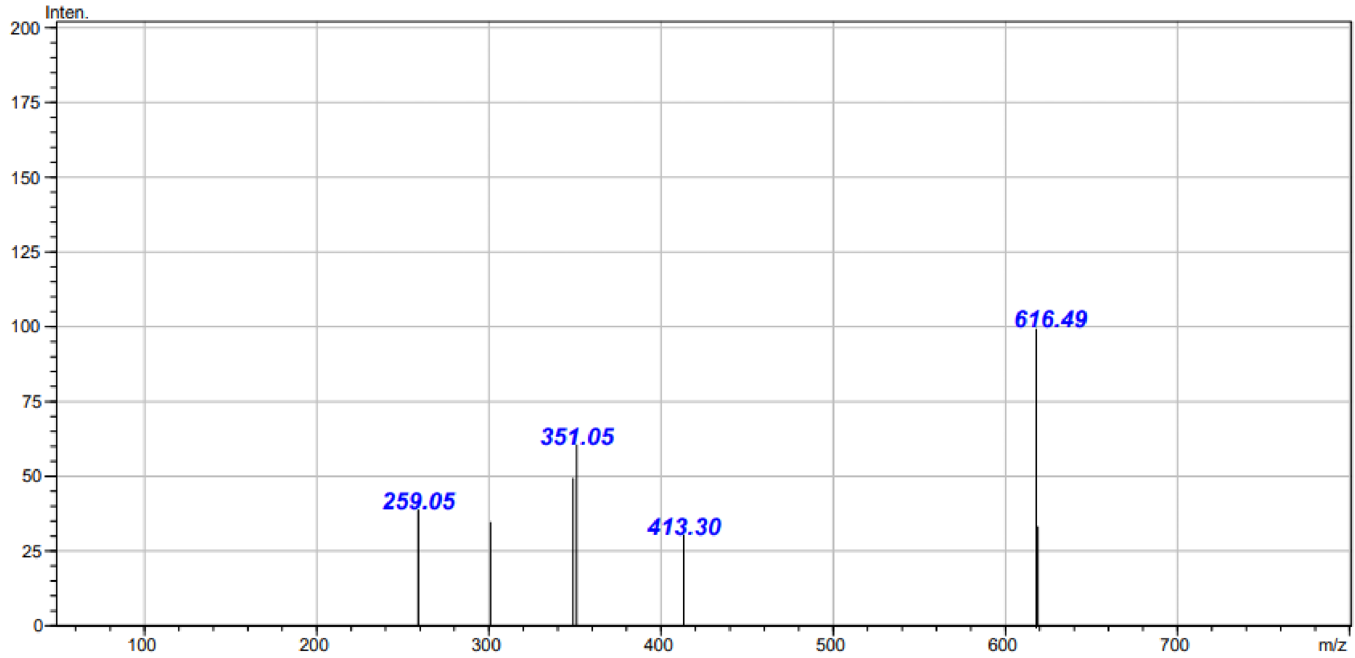

Figure. S10 Compound T7*:* 4-(5-(4-bromophenyl)-3-(2-(4-phenoxybenzylidene) hydrazine-1-carbonyl)-1H-pyrazol-1-yl) benzenesulfonamide. Yellow coloured solid, yield 80%, m.p. 170~ 171°C. 3397 (N-H), 3063 (aromatic N-H), 2998 (C–H aromatic),2840 (aldehyde C – H), 1335 (sulphonamide S=O), 1664 (C=O), 1098 (C-N), 1406 (alkenes C=C) and 689 (C-Br). The ^{1}H NMR (DMSO-d6, 400 MHz) spectra showed δ at 11.81 (s, 1H, CONH), 8.55 (s, 1H, CHN), 7.90 (d, 2H, ArH), 7.72 (t, 3H, ArH), 7.60 (m, 3H, ArH), 7.52 (t, 5H, ArH and SO_2NH_2), 7.42 (d, 2H, ArH), 7.24 (s, 2H, ArH), 7.11 (s, 1H, CH),

6.98 (d, 2H, ArH). ^{13}C NMR δ: 160.24, 157.64, 148.56, 146.74, 145.34, 144.26, 140.15, 135.08, 131.36, 128.08, 127.46, 121.82, 121.24, 118.90, 116.54, 114.80, 108.40. The mass spectra showed a peak at 616.52 where $[M+H]^+$ is 616.49.

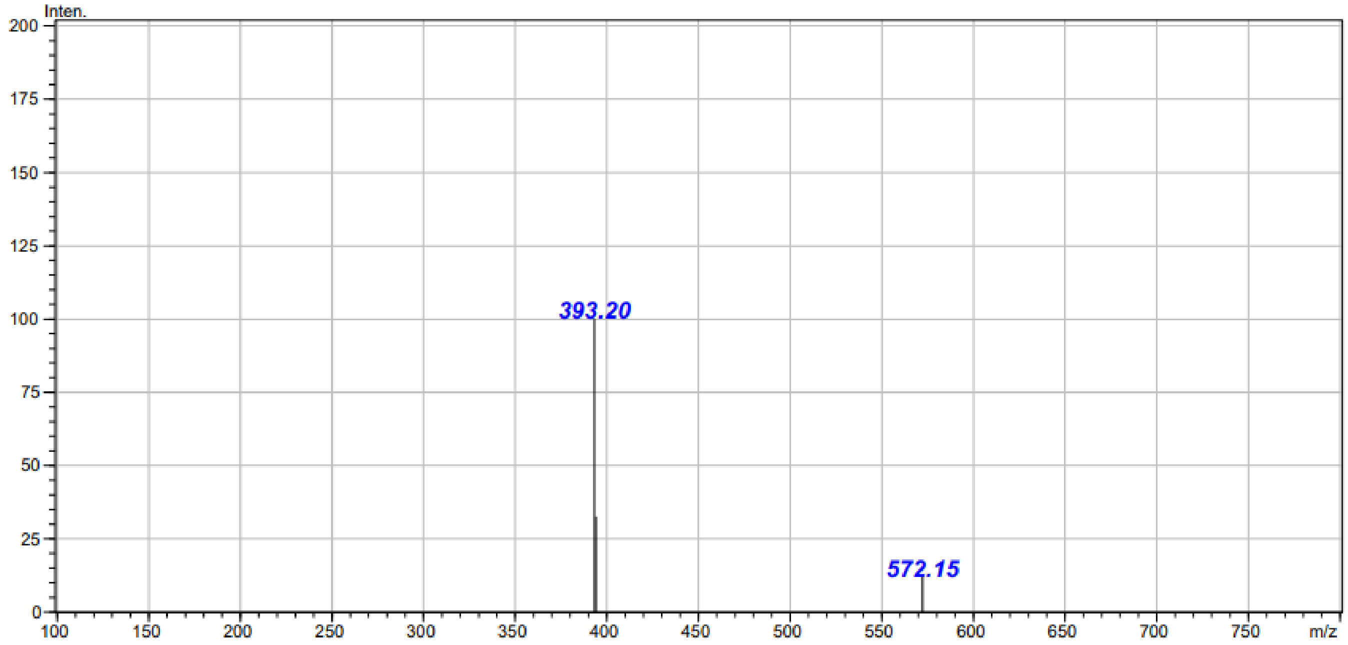

Figure. S11 Compound T8: 4-(5-(4-chlorophenyl)-3-(2-(4-phenoxybenzylidene) hydrazine-1-carbonyl)-1H-pyrazol-1-yl) benzenesulfonamide. Light yellow coloured solid, yield 78%, m.p. 190~ 192°C. 3397 (N-H), 3065 (aromatic N-H), 2998 (C–H aromatic)2363(aldehyde C – H), 1328 (sulphonamide S=O), 1669 (C=O), 1094(C-N),1409 (alkenes C=C) and 834 (C-Cl). The ^{1}H NMR (DMSO-d6, 400 MHz) spectra showed δ at 11.84 (s, 1H, CONH), 8.76 (s, 1H, CHN), 7.88~7.86 (m, 4H, ArH), 7.60 (m, 3H, ArH), 7.48 (s, 2H, SO_2NH_2), 7.42 (m, 4H, ArH), 7.24 (t, 4H, ArH), 7.16 (s, 1H, CH), 6.98 (d, 2H, ArH). ^{13}C NMR δ: 158.88, 157.60, 148.42, 146.72, 145.28, 144.13, 142.15, 137.08, 129.17, 127.03, 124.60, 123.79, 123.42, 122.36, 116.52, 114.81, 108.87. The mass spectra showed a peak at 572.15 where $[M+H]^+$ is 572.03.

Priya-1

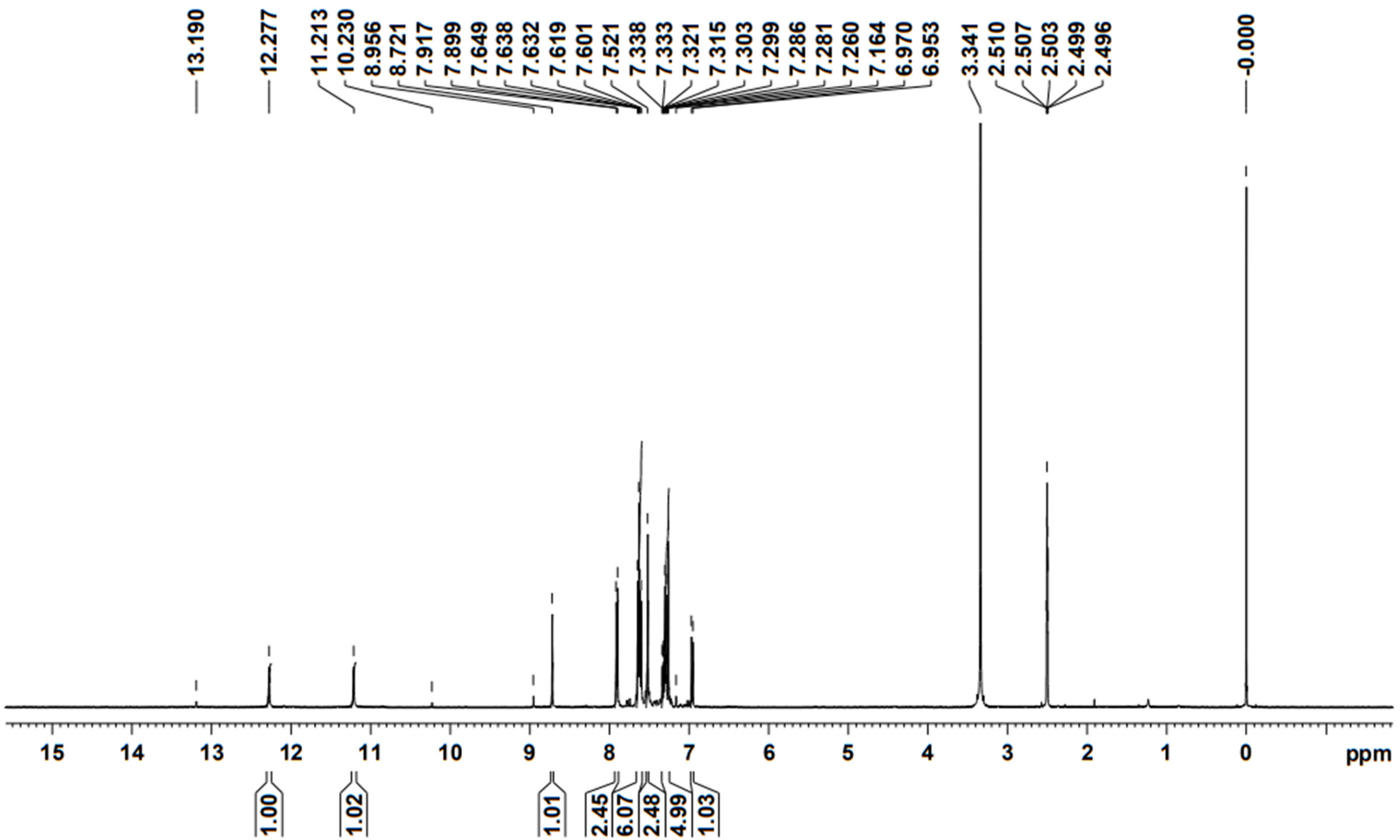

13.190
12.277
11.213
10.230
8.956
8.721
7.917
7.899
7.649
7.638
7.632
7.619
7.601
7.521
7.338
7.333
7.321
7.315
7.303
7.299
7.286
7.281
7.260
7.164
6.970
6.953
3.341
2.510
2.507
2.503
2.499
2.496
-0.000
15
14
13
12
11
10
9
8
7
6
5
4
3
2
1
0
ppm
1.00
1.02
1.01
2.45
6.07
2.48
4.99
1.03

Figure. S12 Compound T9*:* 4-(5-(4-bromophenyl)-3-(2-(4-chloro-2-hydroxybenzylidene) hydrazine-1-carbonyl)-1H-pyrazol-1-yl) benzenesulfonamide. Yellow coloured crystals, yield 84%, m.p. 250~251°C. 3282 (O-H), 3436 (N-H), 3186 (aromatic N-H), 3110 (C– H aromatic),2268(aldehyde C – H), 1343 (sulphonamide S=O), 1672 (C=O), 1143 (C-N), 1402 (alkenes C=C), 607 (C-Br)and 827 (C-Cl). The ^{1}H NMR (DMSO-d6, 400 MHz) spectra showed δ at 12.27 (s, 1H, CONH), 11.21 (s, 1H, OH), 10.23 (s, 1H, CHN), 8.71 (d, 2H, ArH), 7.91~7.89 (m, 2H, ArH), 7.65~7.60 (m, 4H, ArH and SO_2NH_2), 7.52 (d, 2H, ArH), 7.33~7.26 (m, 3H, ArH), 6.97 (s, 1H, CH). ^{13}C NMR δ: 157.75, 156.50, 146.73, 144.33, 144.09, 141.72, 133.18, 132.31, 131.30, 129.14, 128.48, 127.97, 126.48, 123.46, 123.20, 121.26, 118.96, 118.72, 109.77, 40.47. The mass spectra showed a peak at 573.95 where $[M^{+}H]^{+}$ is 574.83.

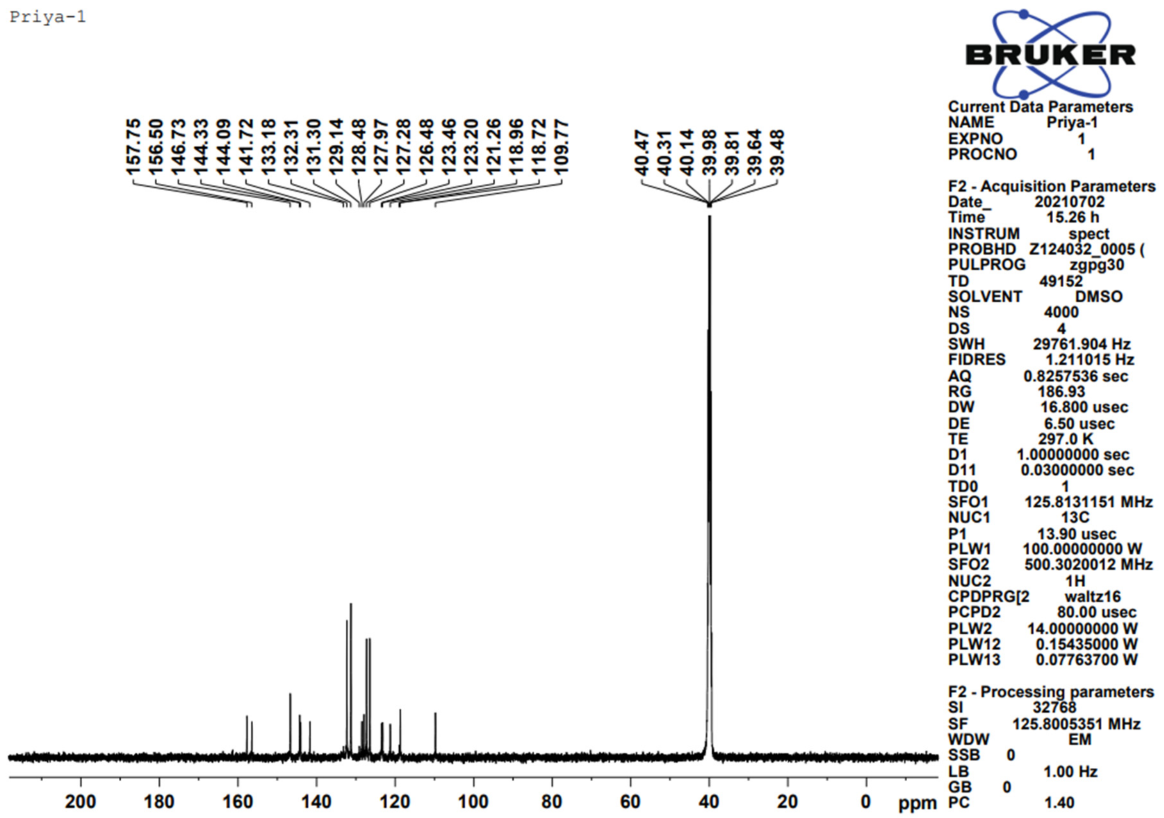

Figure. S13 Compound T10*:* 4-(3-(2-(4-chloro-2-hydroxybenzylidene) hydrazine-1-carbonyl)-5-phenyl-1H pyrazol-1-yl) benzenesulfonamide. Brown coloured solid, yield 55%, m.p. 260~261˚C. 3282 (O-H), 3436 (N-H), 3186 (aromatic N-H), 3110 (C – H aromatic) 2268 (aldehyde C – H), 1343 (sulphonamide S=O), 1672 (C=O), 1143 (C-N), and 1402 (alkenes C=C). The ^{1}H NMR (DMSO-d6, 400 MHz) spectra showed δ at 12.24 (s, 1H, CONH), 11.24 (s, 1H, OH), 8.72 (s, 1H, CHN), 7.91 (d, 2H, ArH), 7.89 (m, 3H, ArH), 7.64 (m, 4H, ArH and SO_2NH_2), 7.52 (m, 3H, ArH), 7.16 (s, 1H, CH), 6.97 (d, 2H, ArH). ^{13}C NMR δ: 157.85, 156.12, 147.74, 145.33, 144.72, 137.18, 132.31, 129.14, 127.04, 126.48, 123.46, 121.26, 118.96, 109.12. The mass spectra showed a peak at 496.05 where $[M^{+}H]^{+}$ is 495.95

Table S1. ADME properties calculated using SwissADME.

Compound	MW g/mol	No. of heavy atoms	HBA	HBD	No. of Violations	No.of rotatable Bonds	miLogP	TPSA (A°)
T1	567.61	41	8	2	1	10	2.59	146.28
T2	567.61	41	8	2	1	10	5.24	146.28
T3	572.03	40	7	2	1	9	3.36	137.05
T4	616.49	40	7	2	1	9	3.45	137.05
T5	658.56	43	7	2	1	10	3.74	137.05
T6	614.11	43	7	2	1	10	3.65	137.05
T7	616.49	40	7	2	1	9	3.45	137.05
T8	572.03	40	7	2	1	9	3.36	137.05
T9	574.83	35	7	3	1	7	2.66	148.05
T10	495.94	34	7	3	0	7	2.09	148.05
Celecoxib	381.37	26	7	1	0	4	2.65	86.36

Table S1A. Structure of best designed compounds

Compound 6 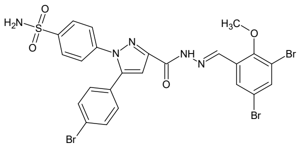	Compound 7 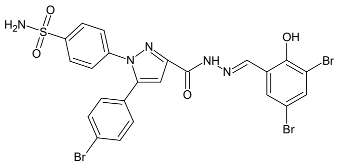
Compound 13 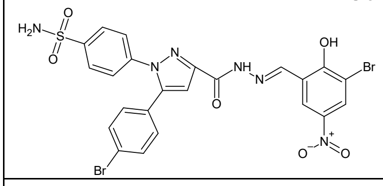	Compound 36 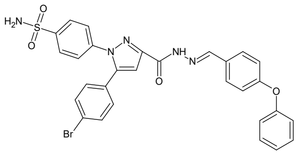
Compound 53 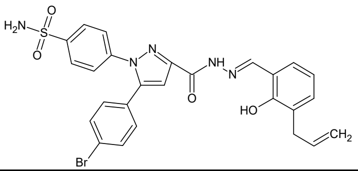	Compound 64 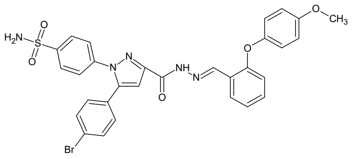
Compound 78 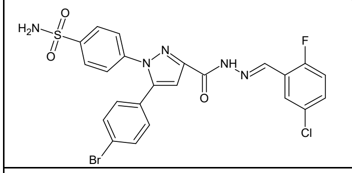	Compound 86 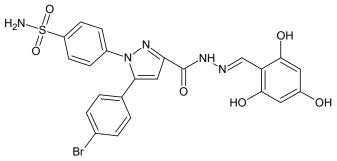
Compound 94	Compound 95

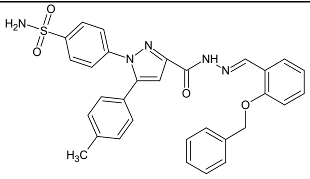	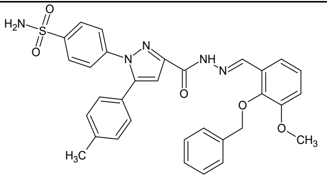
Compound 97<br>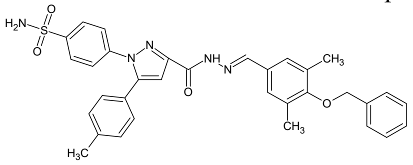	Compound 114<br>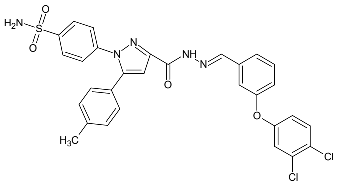
Compound 116<br>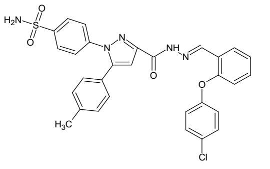	Compound 121<br>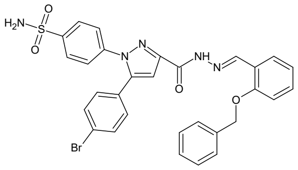
Compound 122<br>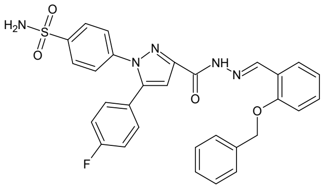	Compound 123<br>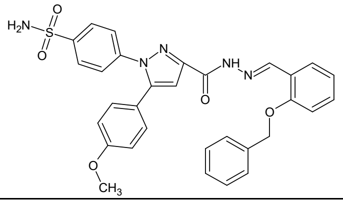
Compound 124<br>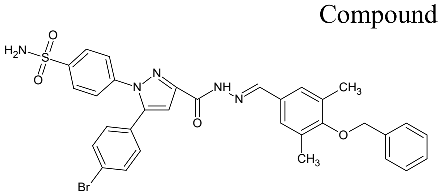	Compound 125<br>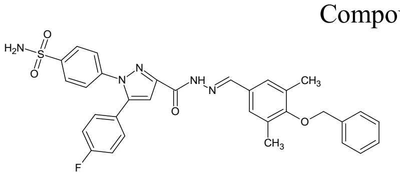
Compound 126<br>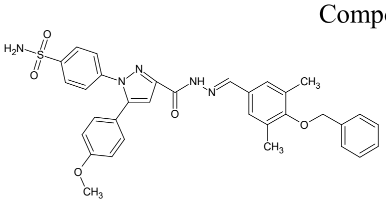	Compound 127<br>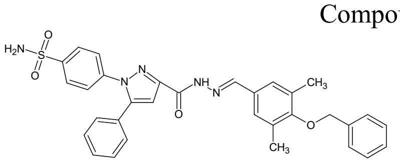
Compound 128<br>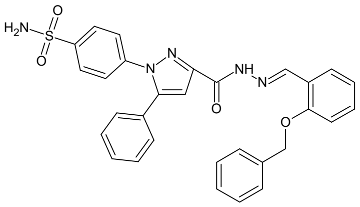	Compound 129<br>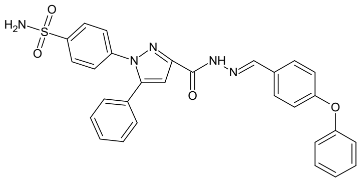
Compound 130	Compound 131

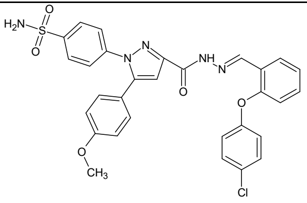	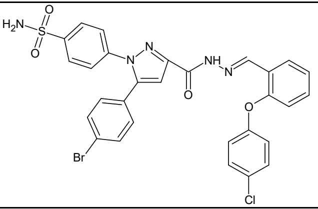
Compound 132 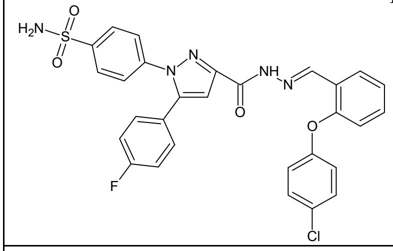	Compound 133 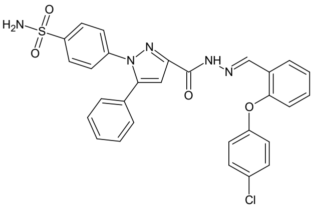
Compound 135 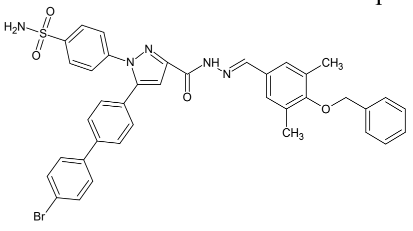	Compound 137 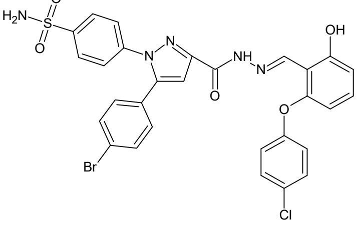
Compound 148 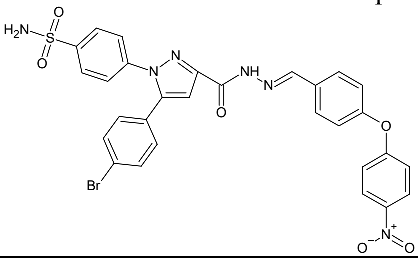	Compound 151 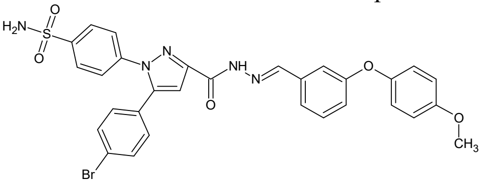
Compound 153 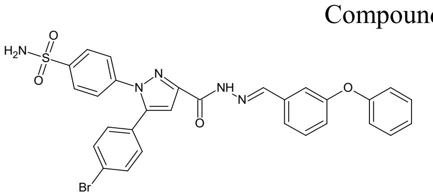	Compound 154 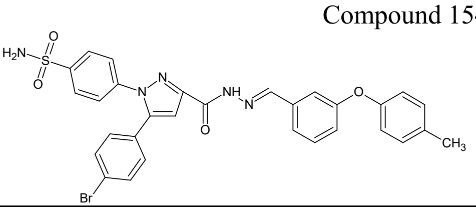
Compound 155 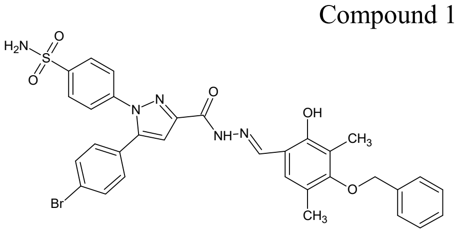	Compound 156 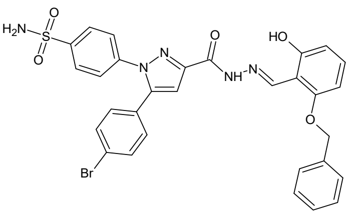

Table S2. Toxicological properties calculated using PROTOX-II (IA indicates inactive and A is active).

	Target				
Compound	Hepatotoxicity	Carcinogenicity	Immunotoxicity	Mutagenicity	Cytotoxicity
T1	IA	IA	A	IA	IA
T2	IA	IA	IA	IA	IA
T3	IA	IA	A	IA	IA
T4	IA	IA	A	IA	IA
T5	A	IA	IA	IA	IA
T6	A	IA	IA	IA	IA
T7	IA	IA	IA	IA	IA
T8	IA	IA	IA	IA	IA
T9	IA	IA	A	IA	IA
T10	IA	IA	IA	IA	IA
Celecoxib	IA	A	IA	IA	IA

Table S3. Toxicity properties using AdmetSAR.

Compound	BBB	HIA	Caco-2 Permeability	AMES Toxicity	Carcinogens	Acute Oral Toxicity	Carcinogenicity
T1	0.5895	0.9945	0.5869	0.6767	0.5661	0.5859	0.6246
T2	0.5895	0.9945	0.5869	0.6767	0.5661	0.5859	0.6246
T3	0.5277	0.9958	0.5751	0.7273	0.5000	0.5625	0.6304
T4	0.5842	0.9939	0.5700	0.7024	0.5288	0.5602	0.6276
T5	0.5617	0.9905	0.5860	0.6226	0.5345	0.5827	0.5990
T6	0.5168	0.9935	0.5872	0.6404	0.5691	0.5904	0.6006
T7	0.5842	0.9939	0.5700	0.7024	0.5288	0.5602	0.6276
T8	0.5277	0.9958	0.5751	0.7273	0.5000	0.5625	0.6304
T9	0.6890	0.9926	0.5788	0.7304	0.5694	0.5797	0.6316
T10	0.7001	0.9892	0.5779	0.7510	0.5817	0.5830	0.6393
Celecoxib	0.9713	0.9878	0.8866	0.7185	0.7905	0.6499	0.7022

Table S4. % COX-2 Inhibition of compound T3.

Sample	Concentration (µg/ml)	% Inhibition
T3	250	85.38 ± 0.73
	100	81.06 ± 0.47
	50	78.94 ± 0.72
	25	76.28 ± 0.70
	12.5	73.95 ± 0.27
	6.25	71.48 ± 0.78
	3.125	66.67 ± 0.96
	1.562	61.82 ± 1.20
	0.781	55.34 ± 1.03

Table S5. % COX-2 Inhibition of compound T5.

Sample	Concentration (µg/ml)	% Inhibition
T5	250	89.35 ± 0.86
	100	88.64 ± 0.86
	50	87.64 ± 1.43
	25	86.71 ± 0.50
	12.5	85.04 ± 2.38
	6.25	84.28 ± 2.50
	3.125	78.03 ± 2.98
	1.562	72.1 ± 6.01
	0.781	65.66 ± 1.05

Table S6. % COX-1 Inhibition of compound T3.

Sample	Concentration (µg/ml)	% Inhibition
T3	250	89.26 ± 0.71
	100	87.94 ± 0.94
	50	85.41 ± 0.12
	25	77.09 ± 0.66
	12.5	73.08 ± 1.76
	6.25	61.14 ± 3.03
	3.125	49.90 ± 2.32
	1.562	24.62 ± 2.13
	0.781	16.22 ± 0.61

Table S7. % COX-1 Inhibition of compound T5

Sample	Concentration (µg/ml)	% Inhibition
T5	250	91.75 ± 2.76
	100	88.49 ± 0.36
	50	84.01 ± 1.77
	25	76.66 ± 1.30
	12.5	72.58 ± 3.09
	6.25	57.14 ± 2.17
	3.125	45.47 ± 1.94
	1.562	21.90 ± 2.60
	0.781	14.39 ± 2.17

Table S8. % cell viability of selected synthetic compounds against A549 cell line

**Concentration µg/ml**	**% cell viability**					
	**T2**	**T3**	**T5**	**T6**	**T9**	**Cisplatin**
**6.25**	95.63	95.77	97.15	97.7	91.72	48.78
**12.5**	81.83	86.16	92.32	86.99	84.82	27.13
**25**	54.02	60.37	72.36	78.85	76.64	15.54
**50**	33.98	46.16	62.43	74.62	66.94	8.64
**100**	18.25	24.18	30.52	63.35	54.57	2.39

Table S9. Cell viability of selected synthetic compounds against HepG2 cell lines

Concentration µg/ml	% cell viability					
	**T2**	**T3**	**T5**	**T6**	**T9**	**Cisplatin**
**6.25**	78.42	84.39	84.40	24.12	85.46	45.30
**12.5**	77.90	84.40	73.66	10.97	84.90	27.32
**25**	71.82	73.65	73.42	2.99	79.30	11.76
**50**	67.10	73.62	54.76	2.87	67.24	11.00
**100**	64.39	54.76	39.32	2.62	54.42	6.66

Table S10. The average values of RMSD, RMSF, and Rgof protein-ligand complexes.

**Compounds**	**RMSD (Å)**	**RMSF (Å)**	**Rg (Å)**
Protein	1.92	0.9	2.02
T3	1.73	1.00	1.99
T5	3.39	1.15	1.87

Table S11. The average number of H-bonds formed throughout the MD simulation

**Compounds**	**The average no. of H-bonds**
T3	1 ± 0.1
T5	1 ± 0.2
